# Expanding the chemistry of borates with functional [BO_2_]^−^ anions

**DOI:** 10.1038/s41467-021-22835-4

**Published:** 2021-05-10

**Authors:** Chunmei Huang, Miriding Mutailipu, Fangfang Zhang, Kent J. Griffith, Cong Hu, Zhihua Yang, John M. Griffin, Kenneth R. Poeppelmeier, Shilie Pan

**Affiliations:** 1 CAS Key Laboratory of Functional Materials and Devices for Special Environments, Urumqi, China; 2 Xinjiang Technical Institute of Physics & Chemistry, CAS, Urumqi, China; 3 Xinjiang Key Laboratory of Electronic Information Materials and Devices, Urumqi, China; 4 Center of Materials Science and Optoelectronics Engineering, University of Chinese Academy of Sciences, Beijing, China; 5 Department of Chemistry, Northwestern University, Evanston, IL USA; 6 Department of Chemistry, Lancaster University, Bailrigg, Lancaster UK

**Keywords:** Solid-state chemistry, Materials for optics

## Abstract

More than 3900 crystalline borates, including borate minerals and synthetic inorganic borates, in addition to a wealth of industrially-important boron-containing glasses, have been discovered and characterized. Of these compounds, 99.9 % contain only the traditional triangular BO_3_ and tetrahedral BO_4_ units, which polymerize into superstructural motifs. Herein, a mixed metal K_5_Ba_2_(B_10_O_17_)_2_(BO_2_) with linear BO_2_ structural units was obtained, pushing the boundaries of structural diversity and providing a direct strategy toward the maximum thresholds of birefringence for optical materials design. ^11^B solid-state nuclear magnetic resonance (NMR) is a ubiquitous tool in the study of glasses and optical materials; here, density functional theory-based NMR crystallography guided the direct characterization of BO_2_ structural units. The full anisotropic shift and quadrupolar tensors of linear BO_2_ were extracted from K_5_Ba_2_(B_10_O_17_)_2_(BO_2_) containing BO_2_, BO_3_, and BO_4_ and serve as guides to the identification of this powerful moiety in future and, potentially, previously-characterized borate minerals, ceramics, and glasses.

## Abstract (web summary)

Borates offer extended structural diversity and promise in diverse applications. Here the authors report a borate with linear BO_2_ units as well as NMR spectroscopy characterization that provides a quantitative basis for identification of BO_2_ units in polycrystalline and non-crystalline samples.

## Introduction

Borates are in the spotlight owing to their extended structural diversity and broad applications in the design of photonic and optoelectronic devices^1–5^, nuclear waste separation and sequestration^6,7^, commercial glasses^8,9^, amorphous oxide catalysts^10,11^, as well as Li-ion^12,13^ and Mg-ion^14,15^ rechargeable batteries. Consequently, considerable effort has been put toward the search for borates with distinctive atomic structures and properties^16–22^. For borate-based nonlinear optical materials, (i) absorption edge energy, (ii) birefringence, and (iii) nonlinear optical coefficient are the three critical parameters. They are mainly affected by the electronic properties of the microscopic B–O basic units, especially for the alkali and/or alkaline earth metal borates (abbreviated as *A*-borates)^23–26^. Anionic group theory provides the basis for the structure–property relationships^27^. That is to say, the limits of the optical properties of borates lie in the capability of the microscopic units to produce a large bandgap, polarizability anisotropy, and hyperpolarizability^18^. Thus, the maximum thresholds of bandgap, birefringence, and nonlinear optical coefficient of *A*-borates comprising BO_3_ and BO_4_ units, are ~150 nm, ~0.07@1064 nm, and ~0.64 pm/V, respectively, assuming all the basic units are in optimally aligned configurations^18,27^. However, compounds with a higher absorption edge energy (lower wavelength), larger birefringence, and larger nonlinear optical coefficient are required to enable smaller optical devices leading to new uses and increased efficiency in industrial and scientific applications. For example, birefringent materials are essential for modulating polarized light and thus underpin critical optical devices such as beam-splitting polarizers for microscopes and isolators, circulators, and interleavers used in fiber optics for telecommunications^18^. The realization of strategies to push the current limitations of *A*-borates is still a grand challenge in the field of optical materials.

Vacant *p* orbitals in boron cause it to behave as an electron pair acceptor to interact with oxygen nucleophiles and form *sp*, *sp*^2^, and *sp*^3^ hybridized chemical bonds to construct linear BO_2_, triangular BO_3_, and tetrahedral BO_4_ basic units. Despite the nearly 300 natural borate minerals, 3000 borate crystals, and extensive glass research on non-crystalline alkali borates, the structure models are almost exclusively built up from the BO_3_ and BO_4_ polyhedra. Even, linear BO_2_ units are rare and have only been observed in apatite-type A_10_(PO_4_)_6–*x*–*y*_(SiO_4_)_*x*_(BO_4_)_*y*_(BO_2_) and Gd_4_(BO_2_)O_5_F^28–31^. It should be noted that the apatite compounds contain atomic disorder and the two isolated Gd_4_(BO_2_)O_5_F crystals are twinned^31^. Thus, among the thousands of borates, there is an absence of studies of borate-based optical materials with BO_2_ units. Structurally, the realization of borates with the BO_2_ functionality will dramatically enrich the structural diversity of this technologically important family of materials and offer a path toward improving the three critical optical parameters in *A*-borates. This basis stimulates the exploration of *A*-borates with BO_2_ configurations and routes toward a more fundamental understanding of the origin of this unique building unit.

Herein, a mixed metal borate K_5_Ba_2_(B_10_O_17_)_2_(BO_2_) with BO_2_ units was obtained. Boron solid-state nuclear magnetic resonance (NMR) spectroscopy has long been a core characterization tool in the study of borate crystals^32,33^ and glasses^34,35^. The resonances ascribed to BO_4_ units are observed from 2 to –4 ppm and are narrow due to their small quadrupolar coupling constants of typically <1 MHz while the resonances ascribed to BO_3_ units appear from 12 to 19 ppm and are broader with quadrupolar coupling constants of 2–3 MHz^34,36,37^. Differences in the number of non-bridging oxygen atoms within a given local coordination can often be determined through careful analysis of ^11^B shielding^33,38^. The ^11^B spectral characteristics of linear BO_2_ observed here in K_5_Ba_2_(B_10_O_17_)_2_(BO_2_) do not follow the chemical shift trend but rather are unique in the anisotropy of the shielding interaction. The ordered, diamagnetic structure enables clear identification of BO_2_ even in the presence of BO_3_ and BO_4_ units. The solid-state NMR signatures of the 11 distinct boron sites in K_5_Ba_2_(B_10_O_17_)_2_(BO_2_) are examined from multifield experimental spectra recorded under static and magic-angle spinning conditions through the support of DFT calculations and multiple-quantum magic-angle spinning (MQMAS) experiments.

K_5_Ba_2_(B_10_O_17_)_2_(BO_2_) expands the frontiers of structural diversity and functionality. Structurally, this is the first compound that contains all three borate motifs simultaneously, namely linear BO_2_, trigonal BO_3_, and tetrahedral BO_4_. The BO_2_ units have the largest polarizability anisotropy, and thus push the maximum thresholds of birefringence in *A*-borates to 0.18@1064 nm, which is much higher than can be attained in compounds with only BO_3_ and BO_4_. Such a large theoretical birefringence from this motif sets a target for the design of optical materials.

## Results and discussion

### Crystal structure of K_5_Ba_2_(B_10_O_17_)_2_(BO_2_)

**Fig. 1:**
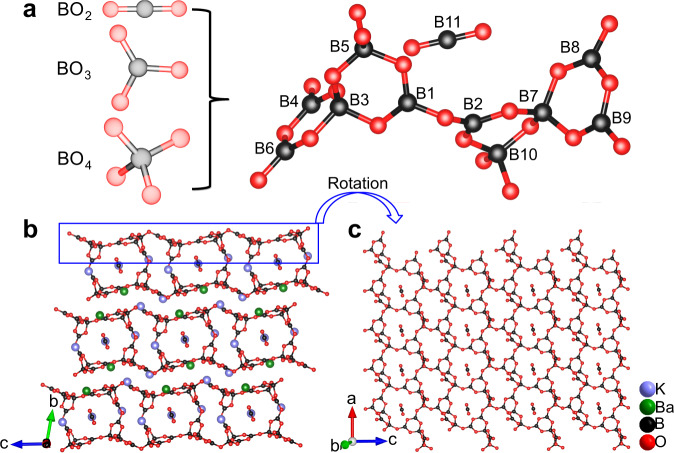
Crystal-structural features of K_5_Ba_2_(B_10_O_17_)_2_(BO_2_). **a** B–O basic units and their relative positions in the asymmetric unit of K_5_Ba_2_(B_10_O_17_)_2_(BO_2_). K_5_Ba_2_[B_10_O_17_]_2_(BO_2_) presents the first case that contains all the three available basic motifs simultaneously, namely BO_2_, BO_3_, and BO_4_ units. **b** Crystal structure of K_5_Ba_2_(B_10_O_17_)_2_(BO_2_) viewed along the *a*-axis. **c** The ^2^[B_5_O_9_]_∞_ infinite layer and isolated [BO_2_]^−^ anions in K_5_Ba_2_(B_10_O_17_)_2_(BO_2_). The adjacent ^2^[B_5_O_9_]_∞_ infinite layers further link to form ^2^[B_10_O_17_]_∞_ double layers and then stack along the [010] direction in the –AAAA– sequence. The atoms in lavender, green, black, and red are K, Ba, B, and O atoms, respectively. All the bonds of K–O and Ba–O are removed for clarity. The O atoms in isolated [BO_2_]^−^ units are non-bridging atoms.

Single crystals of K_5_Ba_2_(B_10_O_17_)_2_(BO_2_) were obtained (Supplementary Fig. 1) by a high-temperature solution method in an open system, and a phase-pure polycrystalline sample was obtained by a stoichiometric solid-state reaction. The crystal structure was solved and refined from single-crystal X-ray diffraction (XRD) data. K_5_Ba_2_(B_10_O_17_)_2_(BO_2_) crystallizes in the triclinic crystal system in space group $P\bar{1}$ (Supplementary Table 1). The crystal structure of K_5_Ba_2_(B_10_O_17_)_2_(BO_2_) features two dimensional (2D) ^2^[B_10_O_17_]_∞_ double layers with K- and Ba-based polyhedra residing in the tunnels and interlayers (Fig. 1). The asymmetric unit of K_5_Ba_2_(B_10_O_17_)_2_(BO_2_) contains three, one, eleven, and eighteen crystallographically independent K, Ba, B, and O atoms, respectively (Supplementary Table 2). With respect to the B–O polyanionic framework, the B(11) atom resides in linear BO_2_ coordination, while B(1, 2, 4, 6, 8, 9) atoms sit in trigonal BO_3_ sites and B(3, 5, 7, 10) have tetrahedral oxygen coordination (Fig. 1a and Supplementary Table 2). The B–O bond lengths in BO_2_ (1.255 Å) are shorter than the mean values of BO_3_ (1.385 Å) and BO_4_ (1.475 Å), respectively, and the O–B–O angle refined to 180°, which conforms to *sp* hybridization. The geometries of BO_3_ and BO_4_ here are consistent with those of reported borates^28–31,39–42^. In K_5_Ba_2_(B_10_O_17_)_2_(BO_2_), three BO_3_ and two BO_4_ units form a B_5_O_11_ ring, which is further linked to form a B_10_O_21_ double ring by sharing a common O(7) atom. The double rings spread out over the *ac-* plane by sharing terminal O atoms to construct a ^2^[B_5_O_9_]_∞_ infinite layer, then adjacent layers further link to form a ^2^[B_10_O_17_]_∞_ double layer (Fig. 1c). The double layers are stacked along the [010] direction in the –AAAA– sequence and held together via electrostatic K–O and Ba–O interactions (Fig. 1b). Topologically, the structural framework of K_5_Ba_2_(B_10_O_17_)_2_(BO_2_) can be simplified to a four-connected unimodal net with the Schläfli symbol {4^8^·6^2^}, in which the B_5_O_11_ single ring is considered as the four-connected node. According to the rules proposed by Xue et al.^2^, the linear BO_2_ unit can be denoted by the letter ‘L’, and the algebraic description of the title compound can be written as {11:^2^_∞_[2 < 3∆+2 T>]&L}.

### Solid-state nuclear magnetic resonance spectroscopy of K_5_Ba_2_(B_10_O_17_)_2_(BO_2_)

**Fig. 2:**
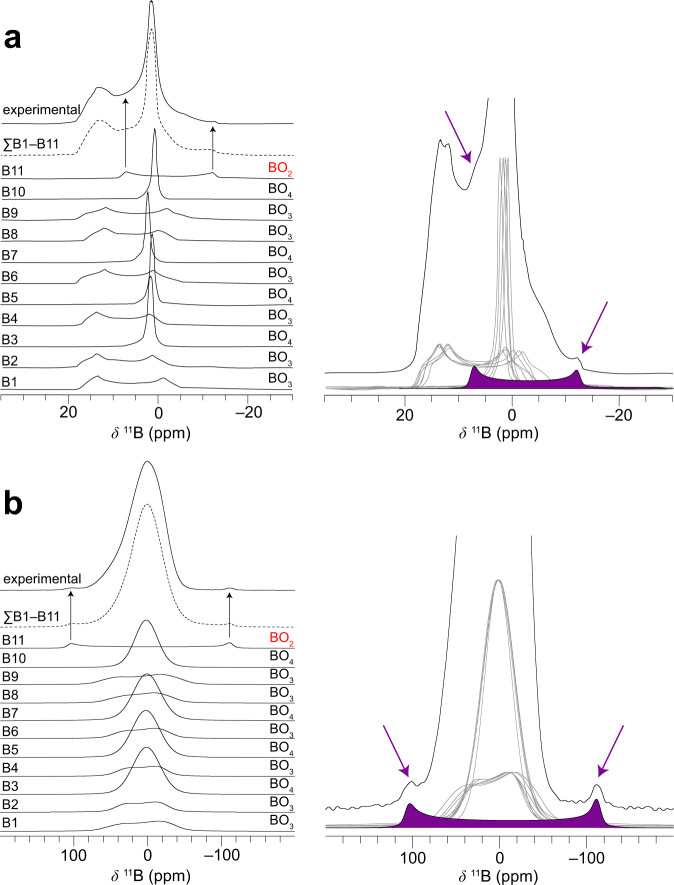
Experimental and simulated ^11^B NMR spectra. (Left) The experimental powder lineshape along with the 11 individual boron sites that comprise the summed simulation are shown for **a** 40 kHz magic-angle spinning and **b** static conditions. The shift and quadrupolar tensors are given in Supplementary Table 5. (Right) Highlighted spectral features of the linear BO_2_ motif and its contribution to the overall lineshape of K_5_Ba_2_(B_10_O_17_)_2_(BO_2_).

Multifield ^11^B static and magic-angle spinning (MAS) NMR spectroscopy, aided by DFT calculations of the chemical shielding and quadrupolar coupling, was used to study the local structure of the boron moieties and to gain fundamental physical insights into the anisotropy of the BO_2_ site (Fig. 2, Supplementary Fig. 2). The signal centered about 1.5 ppm in the MAS spectra (Fig. 2a, Supplementary Fig. 2a) was ascribed to the four overlapped BO_4_ resonances predicted from DFT^32,34^. This low-frequency signal is better resolved at a high magnetic field (Supplementary Fig. 2a) and is resolved into three distinct sites (in 2:1:1 ratio) by multiple-quantum MAS (MQMAS) (Supplementary Figs. 3–4)^43^, confirming the expected number of distinct BO_4_ sites. The low-field (9.4 T) MAS spectrum also revealed a broad shoulder centered about 13 ppm that is characteristic of BO_3_^33^. This feature is nearly baseline-resolved at high field (16.4 T, Supplementary Fig. 2a). The overall MAS lineshapes could almost be reproduced by performing numerical simulations of the shift and quadrupolar parameters of the four BO_4_ and six BO_3_ sites directly from DFT calculations of the K_5_Ba_2_(B_10_O_17_)_2_(BO_2_) XRD structure model. Owing to a large number of adjustable shift and quadrupolar parameters from the ten overlapping distinct BO_4_ and BO_3_ crystallographic sites, all parameters except the isotropic shift were fixed to their calculated values and not refined in the fit (see Supplementary Table 5). However, after calculating the overall lineshape from BO_3_ and BO_4_ contributions, a small feature resembling a powder ‘horn’ line-shape was unaccounted for, which is most distinct at –12 ppm in the low-field MAS data where it does not overlap with other boron sites. Turning to the static ^11^B spectra, the BO_4_ and BO_3_ regions are poorly resolved due to the presence of broadening from chemical shift anisotropy (CSA), dipolar, and second-order quadrupolar interactions. Meanwhile, small but distinct features were observed at *ca*. 100 and –110 ppm at low field (Fig. 2b) and *ca*. 80 ppm at high field (Supplementary Fig. 2b), outside the expected range for known borates. DFT calculations predicted a relatively large *C*_Q_ for BO_2_ but within the same range as BO_3_; the CSA of BO_2_, on the other hand, was calculated to be an order of magnitude larger than those of BO_3_ and BO_4_ units^32^. Finally, the calculated shift of 9 to 10 ppm is intermediate between BO_4_ at lower frequency and BO_3_ at higher frequency. A simulated lineshape based on the DFT-calculated CSA and quadrupolar parameters for the B(11) (BO_2_) site agrees well with the unexplained features in the MAS and static spectra. Allowing the lineshape to be refined against the experimental data yielded an isotropic shift of 13.5(3) ppm, a reduced CSA (Haeberlen convention, see “Methods”) of –123(3) ppm, a quadrupolar coupling constant of 3.31(5) MHz, asymmetries near zero, and coincident shielding and quadrupolar tensors aligned along O–B–O (Fig. 2, Supplementary Figs. 2, 5, and 6, and Supplementary Table 5). The BO_2_ site could not be detected in the MQMAS spectrum due to its low intensity and large *C*_Q_^44,45^; however, the BO_3_ and BO_4_ resonances gave good agreement with the DFT-calculated NMR parameters for the K_5_Ba_2_(B_10_O_17_)_2_(BO_2_) structure model. That the observed *C*_Q_ and CSA were smaller than the calculated values for the BO_2_ B(11) site—and B(11) only—is an indication of partial averaging from dynamics within this isolated (*i.e.*, not covalently bound) moiety^46^. This observation is in agreement with the diffraction-derived atomic displacement parameters of B(11) and O(17) from BO_2_ being approximately a factor of three larger than all the other boron and oxygen atoms for which motion is restricted within the polymeric borate framework.

The intermediate ^11^B shift of BO_2_ is unexpected and does not follow the trend of, *e.g*., ^27^Al in aluminates wherein the shift increases as the coordination number decreases (*δ* ^27^Al: AlO_4_ > AlO_5_ > AlO_6_)^47^. However, the origin of the intermediate shift can be explained by a change in bonding from B–O single bonds in BO_4_ and BO_3_ to B = O double bonds in BO_2_. Note that O=B=O has a formal negative charge on the boron center. The borate NMR picture is consistent with the isoelectronic carbon analogues: The ^13^C shift of CO_2_ (126–129 ppm) is shielded relative to CO_3_ (163–171 ppm)^48,49^. Moreover, the large CSA of BO_2_ (–123(3) ppm) is analogous to the large CSA of CO_2_ (–210 ppm)^50^, further highlighting the similarities between these two species. Finally, the shift and quadrupolar asymmetry parameters *η*_CSA_ and *η*_Q_, respectively, for the central atom of a linear species with D_∞h_ symmetry should be zero (*δ*_XX_ = *δ*_YY_; *V*_XX_ = *V*_YY_). An *η*_CSA_ of zero was found for solid ^13^CO_2_ within experimental uncertainty^47^. Similarly, *η*_CSA_ and *η*_Q_ for ^11^BO_2_ in K_5_Ba_2_(B_10_O_17_)_2_(BO_2_) were calculated to be slightly above zero due to interactions with neighboring atoms and were observed to be zero or near zero within the experimental uncertainty.

The chemical shift anisotropy is by far the most distinctive NMR feature of the elusive BO_2_ moiety and thus careful experiments are required to take advantage of this characteristic. Acquiring high signal-to-noise and performing measurements at multiple magnetic field strengths is useful to differentiate line broadening arising from CSA versus the quadrupolar interaction. Furthermore, magic-angle spinning effectively averages CSA but only partially averages second-order quadrupolar interactions, so it is useful to compare static and MAS spectra. The ^11^B parameters of the BO_2_ site are consistent between considerations of symmetry and bonding, DFT calculations, and experimental lineshapes across multiple magnetic field strengths and MAS/static conditions. It was possible to identify the linear BO_2_ unit in K_5_Ba_2_(B_10_O_17_)_2_(BO_2_) although it only comprises 1/21 of the boron atoms in the structure and thus 5 % of the integrated intensity, which is distributed over a broad frequency range. Extending these principles beyond this compound, solid-state ^11^B NMR, as well as ^17^O NMR (see Supplementary NMR Discussion in the Supporting Information), may serve as powerful tools for the identification of BO_2_ groups in borates even in the absence of atomically ordered single crystals.

### Spectral analysis and optical properties combining theory and experiment

**Fig. 3:**
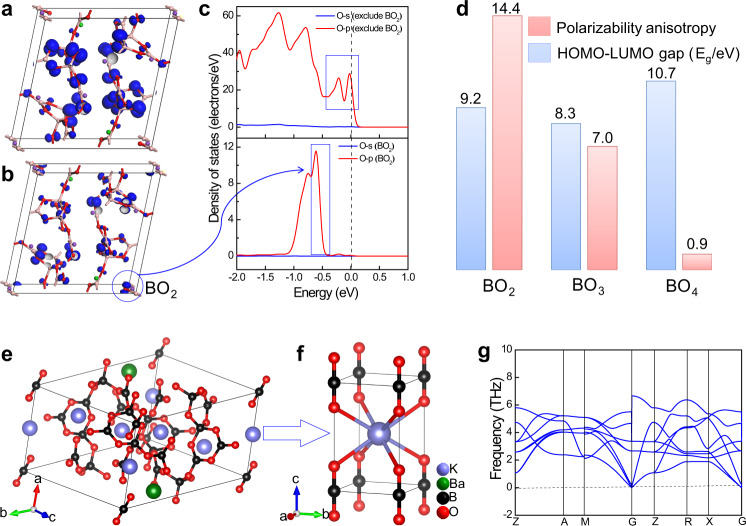
The structure-property relationships of K_5_Ba_2_(B_10_O_17_)_2_(BO_2_). **a**–**c** The structure-property relationships of K_5_Ba_2_(B_10_O_17_)_2_(BO_2_). Frontier orbitals (isosurface) and partial density of states of O in K_5_Ba_2_(B_10_O_17_)_2_(BO_2_). **d** Calculated HOMO–LUMO gap (E_g_) and polarizability anisotropy of B(11)O_2_, B(1)O_3_, and B(3)O_4_ units in K_5_Ba_2_(B_10_O_17_)_2_(BO_2_). The polarizability anisotropy of BO_2_ units is even larger than those of BO_3_ and BO_4_ units. Thus, larger birefringence, as determined by polarizability anisotropy, can be expected in BO_2_-containing borates. **e**, **f** Theoretical structural model of K(BO_2_) with solely BO_2_ units derived from K_5_Ba_2_(B_10_O_17_)_2_(BO_2_). **g** The phonon dispersion of K(BO_2_). There are no soft modes at any wave vectors and none of the imaginary phonon modes are observed, demonstrating that K(BO_2_) is dynamically stable.

The infrared spectrum measurement and vibrational modes calculation of K_5_Ba_2_(B_10_O_17_)_2_(BO_2_) were respectively performed to further confirm the coordination environment of the B atoms. Clearly, the experimental infrared spectrum matches well with the first-principles calculations (Supplementary Fig. 7). In the upper range of both spectra (4000–2500 cm^–1^), no vibrational bands caused by OH^−^ or H_2_O were detectable, which is consistent with the results concluded from the single-crystal data. With respect to the linear BO_2_ units, the weak peak at 2009 cm^–1^ and the strong peak at 1938 cm^–1^ are assigned to the ^10^B–O and ^11^B–O stretch in the linear B(11)O_2_ units^29^. K_5_Ba_2_(B_10_O_17_)_2_(BO_2_) is stable up to 732 °C (see TG–DSC curves and powder XRD patterns, Supplementary Fig. 8), thus sizable single crystals should be grown below this decomposition temperature. The UV−vis−NIR spectrum measured from a polycrystalline sample shows that the reflectance at 190 nm is still about 16.5% and thus its UV cutoff edge is shorter than 190 nm (corresponding to a bandgap larger than 6.52 eV) (Supplementary Fig. 9). Theoretical analysis indicates that K_5_Ba_2_(B_10_O_17_)_2_(BO_2_) has an indirect bandgap of 6.21 eV when calculated with a hybrid (HSE06) functional. With pure DFT, the predicted band gap is only 4.61 eV (Supplementary Fig. 10). This well-known underestimation mainly results from the approximation of exchange-correlation energy under DFT methods; nevertheless, it is useful for qualitative analysis^51^. The electronic transitions among the states near the Fermi level were analyzed. The top of the valence band is mainly composed of O 2*p* orbitals (Supplementary Fig. 11). The O 2*p* orbitals from the linear BO_2_ units are located deeper in the valence band than those in the ^2^[B_10_O_17_]_∞_ framework (Fig. 3a–c). In order to further probe the effect of the BO_2_ units on the electronic structure, the polarizability anisotropy and HOMO–LUMO bandgap were calculated for BO_2_, BO_3_, and BO_4_ units (Fig. 3d). The HOMO–LUMO bandgap of BO_2_ is larger than that of BO_3_ but smaller than that of BO_4_. More importantly, the polarizability anisotropy of BO_2_ is even larger than those of BO_3_ and BO_4_. Thus, larger birefringence, as determined by polarizability anisotropy, can be expected in BO_2_-containing borates. Structurally, the linear BO_2_ units can replace the position of halide ions in the related apatite structure^28–31^. In order to probe the structure–function relationship of BO_2_ units on the optical properties, BO_2_ was computationally substituted for halide ions (Cl^–^_,_ Br^–^) to generate the isostructural halides of K_5_Ba_2_(B_10_O_17_)_2_(BO_2_), namely, K_5_Ba_2_(B_10_O_17_)_2_Cl, and K_5_Ba_2_(B_10_O_17_)_2_Br. Results show that the halides exhibit similar bandgaps (Supplementary Figs. 10 and 12) but smaller birefringence (0.057@1064 nm) than K_5_Ba_2_(B_10_O_17_)_2_(BO_2_) (0.062@1064 nm) (Supplementary Fig. 13). The birefringence enhancement from BO_2_ units is small due to the low density (1/21) of the boron sites in K_5_Ba_2_(B_10_O_17_)_2_(BO_2_). Facing this, we postulate that a borate with a high density of BO_2_ units would be a suitable candidate for a material with a wide bandgap and large birefringence. To test this hypothesis, a structure comprising solely BO_2_ units was investigated: K(BO_2_) (Figs. 3e, 3f). In the phonon spectrum of K(BO_2_), there are no soft modes at any wave vectors and no imaginary phonon modes are observed (Fig. 3g), demonstrating that K(BO_2_) is dynamically stable. The bandgap is 6.11 eV (HSE06 hybrid functional) and the birefringence is 0.18@1064 nm. This birefringence is larger than that of any previously reported *A*-borate, including Ca(BO_2_)_2_ (0.124@1064 nm) comprising infinite chains of co-planar BO_3_ trigonal rings^52^. The predicted birefringence in K(BO_2_) would lead to higher-efficiency beam splitting in light polarization processes across telecommunications and scientific instrumentation applications and provide enhanced ability to create and control polarized light. The BO_2_ functionality could enable optical capabilities, specifically in the rarely accessible deep UV range, and thus sets a target for synthetic crystal growth.

In summary, a mixed metal borate K_5_Ba_2_(B_10_O_17_)_2_(BO_2_) with linear BO_2_ units was obtained successfully. It also presents as the first compound that contains all three available B–O bonding motifs: BO_2_, BO_3_, and BO_4_ units. Solid-state ^11^B NMR spectroscopy unequivocally identified the nature of the BO_2_ units in K_5_Ba_2_(B_10_O_17_)_2_(BO_2_) and the experimental and calculated NMR parameters provide a quantitative basis for the future identification of BO_2_ units in polycrystalline and non-crystalline samples. The beneficial role of BO_2_ units on the bandgap and birefringence in K_5_Ba_2_(B_10_O_17_)_2_(BO_2_) were discussed and a model, K(BO_2_), with only BO_2_ units was designed and analyzed theoretically to motivate future directions for deep UV optical materials. Aligned BO_2_ units with the highest polarizability anisotropy push the maximum thresholds of birefringence in *A*-borates to 0.18@1064 nm—far beyond the birefringence limit with only BO_3_ and BO_4_. K_5_Ba_2_(B_10_O_17_)_2_(BO_2_) expands the frontiers of complex borate chemistry while the unique properties and spectroscopic characterization of the elusive BO_2_ unit motivate and provide a methodology for its broader study.

## Methods

### Synthesis and crystal growth

KNO_3_ (aladdin, 99.5%), Ba(NO_3_)_2_ (aladdin, 99.5%), K_2_CO_3_ (aladdin, 99.0%), B_2_O_3_ (aladdin, 99.9%) and H_3_BO_3_ (aladdin, 99.5%) were used as received. Single crystals of K_5_Ba_2_(B_10_O_17_)_2_(BO_2_) were grown by the high-temperature solution method. A mixture of KNO_3_ (0.379 g, 3.75 mmol), Ba(NO_3_)_2_ (0.392 g, 1.50 mmol), and B_2_O_3_ (0.548 g, 7.87 mmol) were loaded into a platinum crucible. The crucible was heated to 830 °C in 10 h, and held at this temperature for 17 h, and then cooled to 30 °C with a rate of 1.5 °C/h. Colorless and block-shaped crystals of K_5_Ba_2_(B_10_O_17_)_2_(BO_2_) were obtained and manually picked out from the crucible for structural characterization (Supplementary Fig. 1). Polycrystalline samples of K_5_Ba_2_(B_10_O_17_)_2_(BO_2_) can also be obtained by the lower temperature through solid-state reaction based on the following reaction: 2.5K_2_CO_3_ + 2Ba(NO_3_)_2_ + 21H_3_BO_3_ → K_5_Ba_2_(B_10_O_17_)_2_(BO_2_) + 2.5CO_2_↑ + 4NO_2_↑ + 31.5H_2_O↑. A mixture of K_2_CO_3_, Ba(NO_3_)_2_, and H_3_BO_3_ was preheated at 200 °C for 10 h. After that, the temperature was gradually raised to 700 °C with several intermediate grindings and mixings, and then held at a selected temperature for 24 h. The powder XRD pattern of the polycrystalline samples matches well with the one calculated from single-crystal XRD analysis except for some preferred orientation peaks.

### Characterization

Single-crystal XRD data were collected on a Bruker SMART APEX II 4 K CCD diffractometer using Mo Kα radiation (*λ* = 0.71073 Å) at room temperature. Data integration, cell refinement, and absorption corrections were carried out with the program SAINT^53^. The structure was solved by direct methods and refined on *F*^2^ by full-matrix least-squares techniques using the program suite SHELXTL^54^. Solutions were checked for missed symmetry using PLATON. The crystal data give *R*_int_ = 0.0249 and the structure solution parameters are *R*_1_ = 0.0340, *wR*_2_ = 0.0812, GOF = 1.077. Powder XRD data were collected with a Bruker D2 PHASER diffractometer (Cu Kα radiation with *λ* = 1.5418 Å, 2*θ* = 5–70°, scan step width = 0.02 °, and counting time = 1 s/step). During the structure analysis, we found that K(3) and Ba(1) can also be set to share the same sites with the corresponding Ba and K (occupancies of about 97 and 92%, respectively.). But, it has no effect on the B-O framework, thus, in order to build an ideal model to study the BO_2_ units, a disorder-free model was considered. Thermal gravimetric (TG) and differential scanning calorimetry (DSC) measurements were carried out on a simultaneous NETZSCH STA 449 F3 thermal analyzer instrument in a flowing N_2_ atmosphere. The sample was placed in Pt crucible, heated from 40 to 900 °C at a rate of 5 °C/min. Infrared spectroscopy was carried out on a Shimadzu IR Affinity-1 Fourier transform infrared spectrometer in the 400–4000 cm^–1^ range. UV–vis–NIR diffuse-reflectance spectroscopy data in the wavelength range of 190–2600 nm were recorded at room temperature using a powder sample of K_5_Ba_2_(B_10_O_17_)_2_(BO_2_) on a Shimadzu SolidSpec-3700DUV spectrophotometer. ^11^B MAS NMR experiments were performed in static magnetic fields of 9.4 and 16.4 T on Bruker Avance spectrometers at ^11^B Larmor frequencies of 128.25 MHz or 224.60 MHz, respectively. MAS ^11^B NMR spectra were recorded with a single non-selective excitation pulse in a 1.6 mm Phoenix probe (9.4 T) or Bruker 4.0 mm probe (16.4 T) using a short pulse length in an effort to obtain quantitative results under quadrupolar nutation. Static spectra were recorded under similar conditions with the exception of a spin-echo pulse sequence at 16.4 T to mitigate probe background. For the MAS spectra, 2400 scans were acquired with a recycle delay of 40 s (9.4 T) and 16 scans with a recycle delay of 0.25 s (16.4 T); for the static spectra, 768 scans were acquired with a recycle delay of 5 s (9.4 T) and 65,536 scans with a recycle delay of 1 s (16.4 T). The measured *T*_1_, integrated over the overlapped resonances from 19 to –2 ppm, was 6.0 s (via saturation recovery) but no difference was seen in the lineshape for recycle delays from 0.02 to 90 s. Experimental details for MQMAS experiments are given in Supplementary Figs. 3 and 4. Lineshape simulations were performed in the Solid Lineshape Analysis program in Bruker TopSpin 3.6.1. ^11^B isotropic shifts were externally referenced to 0.1 M aqueous H_3_BO_3_ at 19.6 ppm^37^. In this work, the Haeberlen convention is used to describe the chemical shift tensor in terms of the isotropic shift (*δ*_iso_), chemical shift anisotropy (*δ*_CSA_, sometimes called the reduced CSA), and shift asymmetry parameter (*η*_CSA_)^55–57^. In terms of the principal components of the shift tensor (*δ*_XX_, *δ*_YY_, and *δ*_ZZ_):

1 $${\delta }_{{iso}}=\frac{{\delta }_{{XX}}+{\delta }_{{YY}}+{\delta }_{{ZZ}}}{{\delta }_{{iso}}}$$

2 $${\delta }_{{CSA}}={\delta }_{{ZZ}}-{\delta }_{{iso}}$$

3 $${\eta }_{{CSA}}=\frac{{\delta }_{{YY}}-{\delta }_{{XX}}}{{\delta }_{{ZZ}}-{\delta }_{{iso}}}$$

and the principal components are ordered such that $\left|{\delta }_{{{ZZ}}}-{\delta }_{{{iso}}}\right|\ge \left|{\delta }_{{{XX}}}-{\delta }_{{{iso}}}\right| > \left|{\delta }_{{{YY}}}-{\delta }_{{{iso}}}\right|$. The quadrupolar tensor is described by the nuclear quadrupolar coupling constant (*C*_Q_) and quadrupolar asymmetry parameter (*η*_Q_). In terms of the principal components of the electric field gradient (*V*_XX_, *V*_YY_, *V*_ZZ_):

4 $${C}_{Q}=\frac{eQ{V}_{{ZZ}}}{h}$$

5 $${\eta }_{Q}=\frac{{V}_{{XX}}-{V}_{{YY}}}{{V}_{{ZZ}}}$$

where *e* is the electric charge, *Q* is the nuclear quadrupole moment, and *h* is Planck’s constant.

### Computational methods

The electronic structure as well as optical property calculations were performed by employing CASTEP^58^, a plane-wave pseudopotential DFT package, with the norm-conserving pseudopotentials (NCP)^59^. The Perdew–Burke–Ernzerhof (PBE) functional within the generalized gradient approximation (GGA) was used.^60^ The plane-wave energy cutoff was set at 850 eV. NMR calculations utilized ultrasoft pseudopotentials generated “on-the-fly” in CASTEP^61,62^. The all-electron magnetic response was calculated with the gauge-including projector-augmented wave (GIPAW) method^63^. Self-consistent field (SCF) calculations were performed with a convergence criterion of 1 × 10^−6^ eV/atom on the total energy. The *k*-point separation was set as 2π × 0.04 Å^–1^ in the Brillouin zone corresponding to primitive cell, resulting in Monkhorst–Pack *k*-point meshes of 4 × 3 × 4. In addition, we also calculated bandgaps adopting the nonlocal exchange functional HSE06 a widely used hybrid functional with relatively high efficiency in calculating bandgaps^64^. The HSE XC functional energy is calculated as follows,

6 $${E}_{xc}^{{\rm{HSE}}}=a{E}_{x}^{{\rm{HF}},{\rm{SR}}}(\omega )+(1-a){E}_{x}^{{\rm{PBE}},{\rm{SR}}}(\omega )+{E}_{x}^{{\rm{PBE}},{\rm{LR}}}(\omega )+{E}_{c}^{{\rm{PBE}}}$$

In HSE06, the parameters are suggested as *a* = 0.25 and *ω* = 0.11 bohr^–1^.

## Supplementary information

Supplementary Information

## Data Availability

All the characterization data and experimental protocols are provided in this article and its Supplementary Information. The X-ray crystallographic coordinates for structures reported in this study have been deposited at the Cambridge Crystallographic Data Centre (CCDC), under deposition numbers 1845847. These data can be obtained free of charge from The Cambridge Crystallographic Data Centre via www.ccdc.cam.ac.uk/data_request/cif.”
