# Supplementary material for: Expanding the chemistry of borates with functional [BO2]− anions
Source: Nat Commun. 2021 May 10;12:2597. doi: 10.1038/s41467-021-22835-4 (PMC8110813; doi:10.1038/s41467-021-22835-4)
Supplement: Supplementary file 1 — Supplementary Information [file 41467_2021_22835_MOESM1_ESM.pdf]

Supplementary Information

Expanding the Chemistry of Borates with Functional $[BO_2]^-$ Anions

Chunmei Huang,[1,2,#] Miriding Mutailipu,[1,2,#] Fangfang Zhang,[1,2,#] Kent J. Griffith,[3] Cong Hu,[1,2] Zhihua Yang,[1,2] John M. Griffin,[4] Kenneth R. Poeppelmeier,[3,*] Shilie Pan[1,2]*

[1]CAS Key Laboratory of Functional Materials and Devices for Special Environments; Xinjiang Technical Institute of Physics & Chemistry, CAS; Xinjiang Key Laboratory of Electronic Information Materials and Devices, 40-1 South Beijing Road, Urumqi 830011, China

[2]Center of Materials Science and Optoelectronics Engineering, University of Chinese Academy of Sciences, Beijing 100049, China

[3]Department of Chemistry, Northwestern University, 2145 Sheridan Road, Evanston, Illinois 60208-3113, United States

[4]Department of Chemistry, Lancaster University, Bailrigg, Lancaster LA1 4YW, United Kingdom

[#]These authors contributed equally

*Corresponding authors, E-mails: krp@northwestern.edu; slpan@ms.xjb.ac.cn

Table of Contents

Supplementary NMR Discussion 3

Table 1. Crystal data and structure refinements of $K_5Ba_2(B_{10}O_{17})_2(BO_2)$ 4

Table 2. Atomic coordinates (x, y, z), equivalent isotropic displacement parameters (U_{eq}), and bond valence sums (BVS) for $K_5Ba_2(B_{10}O_{17})_2(BO_2)$ 5

Table 3. Anisotropic displacement parameters (Å^2) for $K_5Ba_2(B_{10}O_{17})_2(BO_2)$ 6

Table 4. Selected bond lengths (Å) and angles (°) for $K_5Ba_2(B_{10}O_{17})_2(BO_2)$ 7

Table 5. Experimental and calculated boron NMR tensor quantities 10

Table 6. Calculated oxygen-17 NMR tensor quantities 11

Figure. 1. Photograph of $K_5Ba_2(B_{10}O_{17})_2(BO_2)$ crystals 12

Figure. 2. ^{11}B NMR static and MAS spectra of $K_5Ba_2(B_{10}O_{17})_2(BO_2)$ at 16.4 T 13

Figure. 3. ^{11}B MQMAS spectrum of $K_5Ba_2(B_{10}O_{17})_2(BO_2)$ at 9.4 T 14

Figure. 4. Experimental and simulated ^{11}B MQMAS spectra of $K_5Ba_2(B_{10}O_{17})_2(BO_2)$ at 16.4 T 15

Figure. 5. ^{10}B NMR static QCPMG spectrum of $K_5Ba_2(B_{10}O_{17})_2(BO_2)$ at 9.4 T 16

Figure. 6. Visualization of the ^{11}B shielding tensor and ^{17}O quadrupolar coupling tensor overlaid on the partial structure of $K_5Ba_2(B_{10}O_{17})_2(BO_2)$ 17

Figure. 7. Calculated and experimental infrared spectra of $K_5Ba_2(B_{10}O_{17})_2(BO_2)$ 18

Figure. 8. TG–DSC and XRD of $K_5Ba_2(B_{10}O_{17})_2(BO_2)$ 19

Figure. 9. The UV−vis−NIR spectrum of $K_5Ba_2(B_{10}O_{17})_2(BO_2)$ 20

Figure. 10. Band structure of $K_5Ba_2(B_{10}O_{17})_2(BO_2)$ 21

Figure. 11. Density of states of $K_5Ba_2(B_{10}O_{17})_2(BO_2)$ 22

Figure. 12. Band structures of $K_5Ba_2(B_{10}O_{17})_2Cl$ and $K_5Ba_2(B_{10}O_{17})_2Br$ 23

Figure. 13. Calculated birefringence of $K_5Ba_2(B_{10}O_{17})_2X$ (X = BO_2, Cl, Br) 24

References 25

Supplementary Discussion

Boron has a second NMR active isotope, ^{10}B with $I = 3$, but it is not as beneficial for the identification of BO_2 units because ^{10}B has a larger quadrupole moment, lower Larmor frequency, and lower natural abundance than ^{11}B. The integer spin also means that ^{10}B lacks a central transition and thus only the quadrupole-broadened transitions are observed, which, in borates, effectively means that only BO_4 with its small quadrupolar coupling appears[1,2]. The ^{10}B spectra were modeled with the parameters given in Table S5 but showed no distinct spectral features corresponding to BO_2.

Conversely, the NMR calculations reveal that ^{17}O NMR should be quite useful to distinguish BO_2, at least when only borate anions are present. In the case of ^{17}O, oxygen in the BO_2 units should exhibit narrow, low-frequency signals as they are characterized by a calculated isotropic shift and quadrupolar coupling constant of –9 ppm and 1.7 MHz, respectively, compared to the 17 other unique oxygen sites with computed shifts from 24–67 ppm and quadrupolar coupling constants of 4.4–5.9 MHz (Supplementary Table 6, Supplementary Figure. 5).

Supplementary Table 1. Crystal data and structure refinements of $K_5Ba_2(B_{10}O_{17})_2(BO_2)$.

Empirical formula	$K_5Ba_2(B_{10}O_{17})_2(BO_2)$	
Formula weight	1273.19	
Wavelength (Å)	0.71073	
Temperature (K)	143(2)	
Crystal system	Triclinic	
Space group	$P\bar{1}$	
Unit cell dimensions	a = 6.700(10) Å	α = 101.388(10)°
	b =11.178(2) Å	β = 90.441(10)°
	c =11.213(2) Å	γ =104.168(10)°
Z	1	
Volume (Å^3)	796.89(2)	
Density (calc.) (g·cm^{-3})	2.653	
Absorption coefficient (mm^{-1})	3.245	
F(000)	600	
Crystal size (mm^3)	0.121 × 0.101 × 0.085	
Theta range for data collection	1.920 to 27.512	
Limiting indices	$-8 \leq h \leq 8, -14 \leq k \leq 14, -14 \leq l \leq 14$	
Reflections collected / unique	7631 / 3613 [R_{int} = 0.0249]	
Completeness to θ = 27.512	98.7%	
Data / restraints / parameters	3613/6/292	
Goodness-of-fit on F^2	1.077	
Final R indices [$I > 2\sigma(I)$][a]	R_1 = 0.0340 , wR_2 = 0.0812	
R indices (all data)[a]	R_1 = 0.0381 , wR_2 = 0.0832	
Extinction coefficient	n/a	
Largest diff. peak and hole (e·Å^{-3})	1.636 and –1.427	

[a]$R_1 = \Sigma||F_o| - |F_c||/\Sigma|F_o|$ and $wR_2 = [\Sigma w(F_o^2 - F_c^2)^2/ \Sigma wF_o^4]^{1/2}$ for $F_o^2 > 2\sigma(F_o^2)$.

Supplementary Table 2. Wyckoff labels (*Wyck.*), atomic coordinates (*x*, *y*, *z*), equivalent isotropic displacement parameters ($U_{eq.}$), and bond valence sums (BVS) for $K_5Ba_2(B_{10}O_{17})_2(BO_2)$.

Atoms	*Wyck.*	*x*	*y*	*z*	$U_{eq.}$ (Å^2)	BVS
K(1)	1d	5000	10000	0	71(1)	0.85
K(2)	2i	7866(2)	9205(1)	3703(1)	21(1)	0.90
K(3)	2i	4664(1)	5445(1)	3678(1)	3(1)	1.43
Ba(1)	2i	245(1)	6552(1)	–1234(1)	9(1)	2.39
B(1)	2i	2330(8)	8461(5)	2979(4)	9(1)	3.06
B(2)	2i	2569(7)	10766(4)	3421(4)	9(1)	3.05
B(3)	2i	3825(7)	6948(5)	1678(4)	7(1)	3.02
B(4)	2i	4954(7)	6988(5)	–457(4)	8(1)	3.08
B(5)	2i	155(7)	6329(5)	2207(4)	7(1)	3.02
B(6)	2i	7027(7)	6339(4)	949(4)	8(1)	3.03
B(7)	2i	1238(7)	12677(5)	3743(4)	7(1)	2.99
B(8)	2i	–2284(7)	13075(4)	4074(4)	7(1)	3.01
B(9)	2i	–385(7)	6153(4)	4296(4)	7(1)	3.00
B(10)	2i	4404(7)	12530(4)	2651(4)	6(1)	3.04
B(11)	1a	0	10000	0	23(2)	2.74
O(1)	2i	–925(4)	12605(3)	3324(3)	8(1)	2.04
O(2)	2i	2398(5)	9615(3)	3755(3)	12(1)	2.12
O(3)	2i	1302(5)	11437(3)	4010(3)	10(1)	2.02
O(4)	2i	4066(5)	8169(3)	2560(3)	9(1)	1.98
O(5)	2i	5759(5)	6559(3)	1838(3)	11(1)	2.13
O(6)	2i	3645(5)	7208(3)	431(3)	9(1)	2.08
O(7)	2i	4531(5)	7247(3)	–1542(3)	9(1)	2.14
O(8)	2i	–1214(5)	5962(3)	1060(3)	8(1)	1.97
O(9)	2i	–922(5)	5606(3)	3093(3)	7(1)	1.99
O(10)	2i	6605(5)	6517(3)	–227(3)	11(1)	2.22
O(11)	2i	2567(4)	12999(3)	2783(3)	6(1)	2.11
O(12)	2i	–1683(5)	13544(3)	5305(3)	11(1)	2.11
O(13)	2i	1817(5)	13620(3)	4904(3)	10(1)	2.10
O(14)	2i	–4168(5)	13157(3)	3736(3)	8(1)	2.01
O(15)	2i	3896(5)	11148(3)	2591(3)	10(1)	1.86
O(16)	2i	2077(4)	6007(3)	1913(3)	7(1)	2.02
O(17)	2i	897(6)	9135(4)	–316(4)	33(1)	1.83
O(18)	2i	412(5)	7681(3)	2707(3)	9(1)	1.99

Supplementary Table 3. Anisotropic displacement parameters (Å^2) for $K_5Ba_2(B_{10}O_{17})_2(BO_2)$.

**Atom**	U_{11}	U_{22}	U_{33}	U_{23}	U_{13}	U_{12}
K(1)	14(1)	17(1)	170(3)	–11(1)	–13(1)	6(1)
K(2)	11(1)	30(1)	18(1)	–3(1)	1(1)	3(1)
K(3)	3(1)	3(1)	3(1)	1(1)	0(1)	1(1)
Ba(1)	7(1)	12(1)	8(1)	2(1)	1(1)	3(1)
B(1)	11(2)	7(2)	8(2)	3(2)	0(2)	2(2)
B(2)	8(2)	7(2)	8(2)	0(2)	–1(2)	–1(2)
B(3)	7(1)	7(1)	7(1)	2(1)	0(1)	2(1)
B(4)	7(1)	8(1)	8(1)	1(1)	0(1)	2(1)
B(5)	5(2)	9(2)	8(2)	2(2)	1(2)	2(2)
B(6)	7(2)	7(2)	7(2)	1(2)	0(2)	0(2)
B(7)	6(2)	10(2)	5(2)	2(2)	1(2)	3(2)
B(8)	8(2)	9(2)	6(2)	2(2)	3(2)	2(2)
B(9)	9(2)	7(2)	8(2)	4(2)	2(2)	3(2)
B(10)	6(1)	7(1)	6(1)	2(1)	1(1)	2(1)
B(11)	14(4)	23(4)	28(5)	–3(3)	–3(3)	3(3)
O(1)	5(1)	11(2)	8(1)	3(1)	2(1)	4(1)
O(2)	16(2)	8(2)	10(2)	2(1)	5(1)	3(1)
O(3)	11(2)	10(2)	10(2)	5(1)	5(1)	5(1)
O(4)	5(1)	12(2)	9(2)	–1(1)	0(1)	0(1)
O(5)	7(1)	20(2)	9(2)	5(1)	2(1)	7(1)
O(6)	9(2)	16(2)	6(1)	4(1)	4(1)	7(1)
O(7)	8(1)	16(2)	6(1)	4(1)	3(1)	6(1)
O(8)	7(1)	12(2)	6(1)	2(1)	0(1)	4(1)
O(9)	7(1)	9(1)	6(1)	2(1)	1(1)	2(1)
O(10)	9(2)	22(2)	6(2)	4(1)	4(1)	10(1)
O(11)	5(1)	7(1)	6(1)	2(1)	1(1)	2(1)
O(12)	10(1)	14(1)	10(1)	1(1)	1(1)	4(1)
O(13)	6(1)	13(2)	7(2)	–1(1)	0(1)	2(1)
O(14)	6(1)	12(2)	5(1)	1(1)	0(1)	4(1)
O(15)	11(2)	7(1)	12(2)	3(1)	7(1)	3(1)
O(16)	4(1)	10(1)	7(1)	2(1)	2(1)	4(1)
O(17)	22(2)	23(2)	50(3)	–10(2)	–5(2)	12(2)

O(18)	9(2)	8(2)	12(2)	0(1)	3(1)	5(1)

Supplementary Table 4. Selected bond lengths (Å) and angles (°) for $K_5Ba_2(B_{10}O_{17})_2(BO_2)$.

K(1)-O(17)	2.677(4)	Ba(1)-O(10)#8	2.690(3)	B(5)-O(16)	1.443(5)
K(1)-O(17)#1	2.677(4)	Ba(1)-O(1)#9	2.696(3)	B(5)-O(18)	1.472(6)
K(1)-O(15)	3.108(3)	Ba(1)-O(11)#9	2.753(3)	B(5)-O(9)	1.484(5)
K(1)-O(15)#1	3.108(3)	Ba(1)-O(6)	2.791(3)	B(5)-O(8)	1.502(6)
K(1)-O(7)	3.157(3)	Ba(1)-O(17)	2.791(4)	B(6)-O(5)	1.338(6)
K(1)-O(7)#1	3.157(3)	Ba(1)-O(7)	2.828(3)	B(6)-O(8)#2	1.358(6)
K(1)-O(6)#1	3.164(3)	Ba(1)-O(16)#10	2.850(3)	B(6)-O(10)	1.409(6)
K(1)-O(6)	3.164(3)	Ba(1)-O(8)	2.898(3)	B(7)-O(11)	1.446(5)
K(2)-O(4)	2.717(3)	Ba(1)-O(9)#10	2.989(3)	B(7)-O(3)	1.485(6)
K(2)-O(18)#2	2.786(3)	Ba(1)-O(8)#10	3.077(3)	B(7)-O(13)	1.485(5)
K(2)-O(3)#3	2.882(3)	B(1)-O(4)	1.347(6)	B(7)-O(1)	1.499(5)
K(2)-O(3)#2	2.913(3)	B(1)-O(18)	1.359(6)	B(8)-O(14)	1.345(6)
K(2)-O(2)#3	2.921(3)	B(1)-O(2)	1.396(6)	B(8)-O(1)	1.372(6)
K(2)-O(2)#2	2.956(3)	B(2)-O(15)	1.350(6)	B(8)-O(12)	1.394(6)
K(2)-O(5)	3.253(3)	B(2)-O(3)	1.358(6)	B(9)-O(13)#5	1.353(6)
K(3)-O(5)	2.635(3)	B(2)-O(2)	1.389(6)	B(9)-O(9)	1.371(6)
K(3)-O(12)#5	2.682(3)	B(3)-O(16)	1.435(5)	B(9)-O(12)#5	1.392(6)
K(3)-O(13)#3	2.695(3)	B(3)-O(5)	1.484(5)	B(10)-O(11)	1.449(5)
K(3)-O(11)#6	2.733(3)	B(3)-O(4)	1.491(6)	B(10)-O(7)#1	1.474(5)
K(3)-O(14)#7	2.864(3)	B(3)-O(6)	1.493(6)	B(10)-O(14)#2	1.484(6)
K(3)-O(16)	2.879(3)	B(4)-O(7)	1.348(6)	B(10)-O(15)	1.486(6)
K(3)-O(13)#6	2.989(3)	B(4)-O(6)	1.358(6)	B(11)-O(17)#9	1.255(4)
K(3)-O(9)#2	3.005(3)	B(4)-O(10)	1.379(6)	B(11)-O(17)	1.255(4)
K(3)-O(14)#5	3.072(3)				

O(17)-K(1)-O(17)#1	180	O(10)#8-Ba(1)-O(11)#9	68.17(9)
O(17)-K(1)-O(15)	82.01(11)	O(1)#9-Ba(1)-O(11)#9	51.77(8)
O(17)#1-K(1)-O(15)	97.99(11)	O(10)#8-Ba(1)-O(6)	114.81(9)
O(17)-K(1)-O(15)#1	97.99(11)	O(1)#9-Ba(1)-O(6)	115.39(9)
O(17)#1-K(1)-O(15)#1	82.01(11)	O(11)#9-Ba(1)-O(6)	155.40(9)
O(15)-K(1)-O(15)#1	180	O(10)#8-Ba(1)-O(17)	81.61(11)
O(17)-K(1)-O(7)	78.23(10)	O(1)#9-Ba(1)-O(17)	80.57(12)
O(17)#1-K(1)-O(7)	101.77(10)	O(11)#9-Ba(1)-O(17)	82.33(11)
O(15)-K(1)-O(7)	135.45(8)	O(6)-Ba(1)-O(17)	74.29(11)

O(15)#1-K(1)-O(7)	44.55(8)	O(10)#8-Ba(1)-O(7)	159.75(9)
O(17)-K(1)-O(7)#1	101.77(10)	O(1)#9-Ba(1)-O(7)	70.23(9)
O(17)#1-K(1)-O(7)#1	78.23(10)	O(11)#9-Ba(1)-O(7)	121.61(8)
O(15)-K(1)-O(7)#1	44.55(8)	O(6)-Ba(1)-O(7)	48.32(8)
O(15)#1-K(1)-O(7)#1	135.45(8)	O(17)-Ba(1)-O(7)	82.36(10)
O(7)-K(1)-O(7)#1	180	O(10)#8-Ba(1)-O(16)#10	75.25(9)
O(17)-K(1)-O(6)#1	110.09(11)	O(1)#9-Ba(1)-O(16)#10	106.25(9)
O(17)#1-K(1)-O(6)#1	69.91(11)	O(11)#9-Ba(1)-O(16)#10	84.50(8)
O(15)-K(1)-O(6)#1	86.99(8)	O(6)-Ba(1)-O(16)#10	120.09(9)
O(15)#1-K(1)-O(6)#1	93.01(8)	O(17)-Ba(1)-O(16)#10	156.33(10)
O(7)-K(1)-O(6)#1	137.33(7)	O(7)-Ba(1)-O(16)#10	121.31(9)
O(7)#1-K(1)-O(6)#1	42.67(7)	O(10)#8-Ba(1)-O(8)	48.81(9)
O(17)-K(1)-O(6)	69.91(11)	O(1)#9-Ba(1)-O(8)	166.81(9)
O(17)#1-K(1)-O(6)	110.09(11)	O(11)#9-Ba(1)-O(8)	116.82(8)
O(15)-K(1)-O(6)	93.01(8)	O(6)-Ba(1)-O(8)	72.20(8)
O(15)#1-K(1)-O(6)	86.99(8)	O(17)-Ba(1)-O(8)	91.80(12)
O(7)-K(1)-O(6)	42.67(7)	O(7)-Ba(1)-O(8)	119.67(8)
O(7)#1-K(1)-O(6)	137.33(7)	O(16)#10-Ba(1)-O(8)	76.76(8)
O(6)#1-K(1)-O(6)	180	O(10)#8-Ba(1)-O(9)#10	122.54(9)
O(4)-K(2)-O(18)#2	106.97(10)	O(1)#9-Ba(1)-O(9)#10	73.62(8)
O(4)-K(2)-O(3)#3	118.21(10)	O(11)#9-Ba(1)-O(9)#10	91.77(8)
O(18)#2-K(2)-O(3)#3	86.30(9)	O(6)-Ba(1)-O(9)#10	104.90(8)
O(4)-K(2)-O(3)#2	142.09(10)	O(17)-Ba(1)-O(9)#10	150.95(11)
O(18)#2-K(2)-O(3)#2	90.77(9)	O(7)-Ba(1)-O(9)#10	76.62(8)
O(3)#3-K(2)-O(3)#2	95.64(8)	O(16)#10-Ba(1)-O(9)#10	48.75(8)
O(4)-K(2)-O(2)#3	111.57(10)	O(8)-Ba(1)-O(9)#10	115.96(8)
O(18)#2-K(2)-O(2)#3	129.23(10)	O(10)#8-Ba(1)-O(8)#10	106.15(9)
O(3)#3-K(2)-O(2)#3	46.64(9)	O(1)#9-Ba(1)-O(8)#10	118.61(8)
O(3)#2-K(2)-O(2)#3	78.56(9)	O(11)#9-Ba(1)-O(8)#10	128.92(8)
O(4)-K(2)-O(2)#2	151.05(10)	O(6)-Ba(1)-O(8)#10	74.96(8)
O(18)#2-K(2)-O(2)#2	47.58(9)	O(17)-Ba(1)-O(8)#10	148.66(11)
O(3)#3-K(2)-O(2)#2	78.48(9)	O(7)-Ba(1)-O(8)#10	81.81(8)
O(3)#2-K(2)-O(2)#2	46.09(9)	O(16)#10-Ba(1)-O(8)#10	47.21(8)
O(2)#3-K(2)-O(2)#2	97.09(9)	O(8)-Ba(1)-O(8)#10	73.10(9)
O(4)-K(2)-B(2)#3	107.78(11)	O(9)#10-Ba(1)-O(8)#10	46.71(8)
O(4)-K(2)-O(5)	45.70(9)	O(4)-B(1)-O(18)	124.0(4)
O(18)#2-K(2)-O(5)	61.87(8)	O(4)-B(1)-O(2)	121.1(4)
O(3)#3-K(2)-O(5)	106.33(9)	O(18)-B(1)-O(2)	114.8(4)
O(3)#2-K(2)-O(5)	142.81(9)	O(15)-B(2)-O(3)	125.0(4)
O(2)#3-K(2)-O(5)	137.90(9)	O(15)-B(2)-O(2)	121.5(4)
O(2)#2-K(2)-O(5)	109.02(9)	O(3)-B(2)-O(2)	113.5(4)
		O(16)-B(3)-O(5)	110.5(4)
O(5)-K(3)-O(12)#5	105.30(10)	O(16)-B(3)-O(4)	111.6(3)
O(5)-K(3)-O(13)#3	98.84(10)	O(5)-B(3)-O(4)	105.6(3)

O(12)#5-K(3)-O(13)#3	109.76(10)	O(16)-B(3)-O(6)	111.9(4)
O(5)-K(3)-O(11)#6	108.90(10)	O(5)-B(3)-O(6)	109.9(3)
O(12)#5-K(3)-O(11)#6	102.10(10)	O(4)-B(3)-O(6)	107.1(3)
O(13)#3-K(3)-O(11)#6	129.93(9)	O(7)-B(4)-O(6)	116.4(4)
O(5)-K(3)-O(14)#7	118.26(9)	O(7)-B(4)-O(10)	123.5(4)
O(12)#5-K(3)-O(14)#7	133.50(10)	O(6)-B(4)-O(10)	120.1(4)
O(13)#3-K(3)-O(14)#7	80.07(9)	O(16)-B(5)-O(18)	113.2(4)
O(11)#6-K(3)-O(14)#7	50.23(9)	O(16)-B(5)-O(9)	111.1(4)
O(5)-K(3)-O(16)	51.35(9)	O(18)-B(5)-O(9)	108.5(3)
O(12)#5-K(3)-O(16)	67.26(9)	O(16)-B(5)-O(8)	108.0(3)
O(13)#3-K(3)-O(16)	143.40(9)	O(18)-B(5)-O(8)	108.6(3)
O(11)#6-K(3)-O(16)	84.32(9)	O(9)-B(5)-O(8)	107.3(3)
O(14)#7-K(3)-O(16)	129.94(9)	O(5)-B(6)-O(8)#2	126.1(4)
O(5)-K(3)-O(13)#6	153.36(9)	O(5)-B(6)-O(10)	120.2(4)
O(12)#5-K(3)-O(13)#6	69.59(9)	O(8)#2-B(6)-O(10)	113.7(4)
O(13)#3-K(3)-O(13)#6	107.53(8)	O(11)-B(7)-O(3)	111.9(3)
O(11)#6-K(3)-O(13)#6	50.11(8)	O(11)-B(7)-O(13)	112.3(3)
O(14)#7-K(3)-O(13)#6	64.22(9)	O(3)-B(7)-O(13)	107.0(3)
O(16)-K(3)-O(13)#6	105.14(9)	O(11)-B(7)-O(1)	107.7(3)
O(5)-K(3)-O(9)#2	66.95(9)	O(3)-B(7)-O(1)	108.4(3)
O(12)#5-K(3)-O(9)#2	151.93(10)	O(13)-B(7)-O(1)	109.5(3)
O(13)#3-K(3)-O(9)#2	48.96(8)	O(14)-B(8)-O(1)	126.2(4)
O(11)#6-K(3)-O(9)#2	105.92(9)	O(14)-B(8)-O(12)	115.3(4)
O(14)#7-K(3)-O(9)#2	67.47(8)	O(1)-B(8)-O(12)	118.5(4)
O(16)-K(3)-O(9)#2	116.85(8)	O(13)#5-B(9)-O(9)	121.7(4)
O(13)#6-K(3)-O(9)#2	129.44(9)	O(13)#5-B(9)-O(12)#5	119.8(4)
O(5)-K(3)-O(14)#5	124.26(9)	O(9)-B(9)-O(12)#5	118.5(4)
O(12)#5-K(3)-O(14)#5	46.83(9)	O(11)-B(10)-O(7)#1	113.1(4)
O(13)#3-K(3)-O(14)#5	64.94(9)	O(11)-B(10)-O(14)#2	108.3(3)
O(11)#6-K(3)-O(14)#5	122.38(9)	O(7)#1-B(10)-O(14)#2	109.3(3)
O(14)#7-K(3)-O(14)#5	110.96(7)	O(11)-B(10)-O(15)	111.0(3)
O(16)-K(3)-O(14)#5	111.10(8)	O(7)#1-B(10)-O(15)	106.8(3)
O(13)#6-K(3)-O(14)#5	72.35(8)	O(14)#2-B(10)-O(15)	108.1(3)
O(9)#2-K(3)-O(14)#5	113.52(8)	O(17)#9-B(11)-O(17)	180
O(10)#8-Ba(1)-O(1)#9	118.88(9)		

#1 $-x+1, -y+2, -z$ #2 $x+1, y, z$ #3 $-x+1, -y+2, -z+1$
#4 $-x+2, -y+2, -z+1$ #5 $-x, -y+2, -z+1$ #6 $x, y-1, z$
#7 $x+1, y-1, z$ #8 $x-1, y, z$ #9 $-x, -y+2, -z$
#10 $-x, -y+1, -z$ #11 $x, y+1, z$ #12 $x-1, y+1, z$

Supplementary Table 5. Experimental and calculated boron NMR tensor quantities. The same parameters were used to simultaneously fit the static and MAS spectra (Figure. 2). The chemical shift anisotropy (δ_{CSA}), shift asymmetry (η_{CSA}), and Euler angle (β) were determined from the static spectrum because the CSA is averaged out under MAS. Thus, the isotropic shift (δ_{iso}), nuclear quadrupolar coupling constant (C_Q), and quadrupolar asymmetry (η_Q), were the more sensitive parameters in the high-resolution MAS spectrum, essentially free from CSA effects on the lineshape. The Euler angles relating the orientation of the shift and quadrupolar tensors are in the Rose convention. Errors in the final digit are estimated from the quality of fit. Owing to the large number of adjustable shift and quadrupolar parameters from 11 distinct crystallographic sites, many parameters were fixed to their calculated values and not refined in the fit. Empty values in the experimental table below indicate parameters that were not refined. The calculated parameters for B(1)–B(10) from the structure model are thus not necessarily unique, but are reasonably consistent with the experimental spectra. The assignments of individual BO_3 sites are not definitively determined due to the overlapping signals.

Atom	δ_{iso} (ppm)	δ_{CSA} (ppm)	η_{CSA}	C_Q (MHz)	η_Q	α, β, γ (°)
Experimental						
B(1)	20(2)					
B(2)	20(2)					
B(3)	2.1(1)					
B(4)	20(2)					
B(5)	1.9(5)					
B(6)	20(2)					
B(7)	2.8(1)					
B(8)	20(2)					
B(9)	20(2)					
B(10)	1.0(1)					
B(11)	13.5(3)	–123(3)	0.01(5)	3.31(5)	0.03(5)	n/a, 0(2), n/a
Calculated						
B(1)	17.8	11	0.98	3.084	0.20	95, 102, –85
B(2)	17.6	–12	0.85	2.966	0.26	–175, 83, –99
B(3)	1.7	–7	0.25	–0.659	0.67	82, 11, 138
B(4)	15.8	7	0.98	2.882	0.28	–91, 88, –88
B(5)	1.3	–6	0.94	–0.513	0.47	12, 6, –110
B(6)	18.0	–16	0.95	3.005	0.41	171, 92, 90
B(7)	2.5	–6	0.23	–0.527	0.74	87, 23, –20
B(8)	16.9	12	0.94	2.987	0.29	–79, 92, 87
B(9)	20.0	11	0.52	3.257	0.25	–79, 79, –100
B(10)	0.6	–5	0.25	–0.421	0.95	–1, 163, 13

B(11)	9.4	–135	0.01	–4.483	0.03	58, 0, –61

Supplementary Table 6. Calculated oxygen-17 NMR tensor quantities. The oxygen atoms corresponding to BO_2 are O(17). δ_{iso} calculated from $\delta_{iso} = \sigma_{reference} - \sigma_{iso}$ with σ_{iso} of 215.8 ppm according from Middlemiss *et al.*[3]

Atom	δ_{iso} (ppm)	δ_{CSA} (ppm)	η_{CSA}	C_Q (MHz)	η_Q	α, β, γ (°)
Calculated						
O(1)	62	–92	0.89	–5.0	0.14	82, 90, 140
O(2)	29	72	0.31	5.9	0.92	–130, 165, –37
O(3)	61	–83	0.83	–4.5	0.08	–99, 91, –72
O(4)	60	–89	0.92	–4.4	0.21	132, 85, –115
O(5)	50	–88	0.99	–5.0	0.23	94, 91, 57
O(6)	67	–88	0.51	–4.7	0.18	–72, 94, 122
O(7)	52	–67	0.57	–5.7	0.43	–104, 92, 80
O(8)	57	–64	0.91	–5.1	0.57	–116, 107, –98
O(9)	40	62	0.78	5.6	1.00	–40, 23, –23
O(10)	56	–114	0.23	–4.6	0.34	–90, 91, 116
O(11)	24	49	0.58	–5.7	0.79	–90, 18, 114
O(12)	61	–115	0.45	–4.6	0.55	–100, 95, –83
O(13)	62	–94	0.92	–4.9	0.47	–58, 99, 117
O(14)	48	74	0.71	–5.7	0.41	163, 81, 84
O(15)	51	–91	0.83	–4.7	0.55	–48, 100, 112
O(16)	24	–24	0.73	–5.8	0.81	–24, 85, 161
O(17)	–9	–132	0.28	–1.7	0.40	–77, 1, –125
O(18)	57	–91	0.75	–4.6	0.28	51, 98, –109

Supplementary Figure 1. Photograph of $K_5Ba_2(B_{10}O_{17})_2(BO_2)$ crystals. The upper left image is a microscopic image of a submillimeter-sized crystal used to determine the structure of a single crystal. Thin orange grid squares are 1 mm in length.

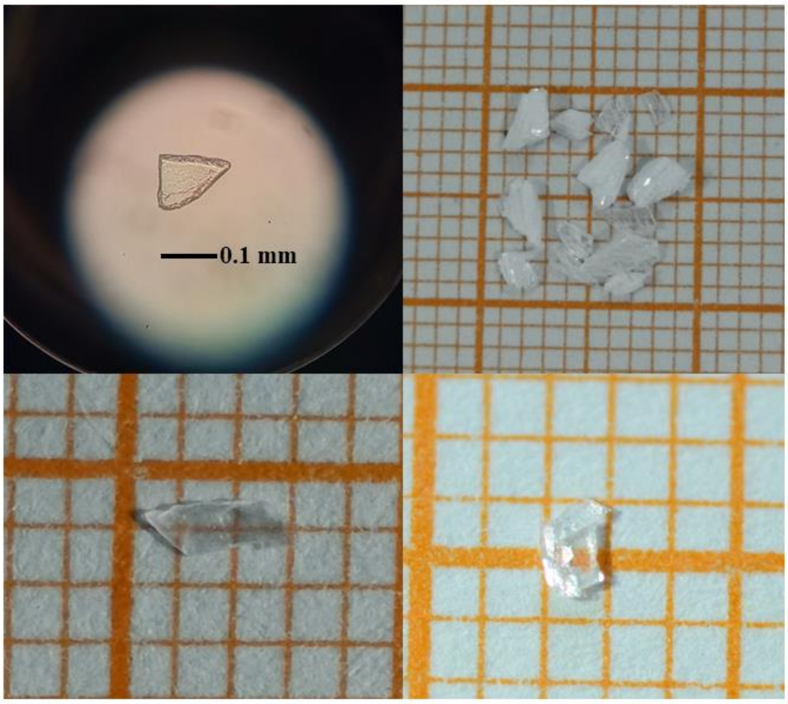

Supplementary Figure 2. ^{11}B NMR static and MAS spectra of $K_5Ba_2(B_{10}O_{17})_2(BO_2)$ at 16.4 T. **a** 12.5 kHz magic-angle spinning and **b** static conditions. The superimposed simulated lineshapes show the 11 individual boron sites that comprise the summed fit. The shift and quadrupolar tensors values in these simulated sites are the same as those in Figure 2 in the main text (and given in Supplementary Table 5). (Right) Highlighted spectral features of the linear BO_2 motif and its contribution to the overall lineshape of $K_5Ba_2(B_{10}O_{17})_2(BO_2)$. The reduced relative intensity of the BO_2 feature in the experimental spectrum compared to the simulation may be due to differences in nutation rate between the BO_2, BO_3, and BO_4 resonances (due to the larger quadrupolar interaction of the BO_2 species).

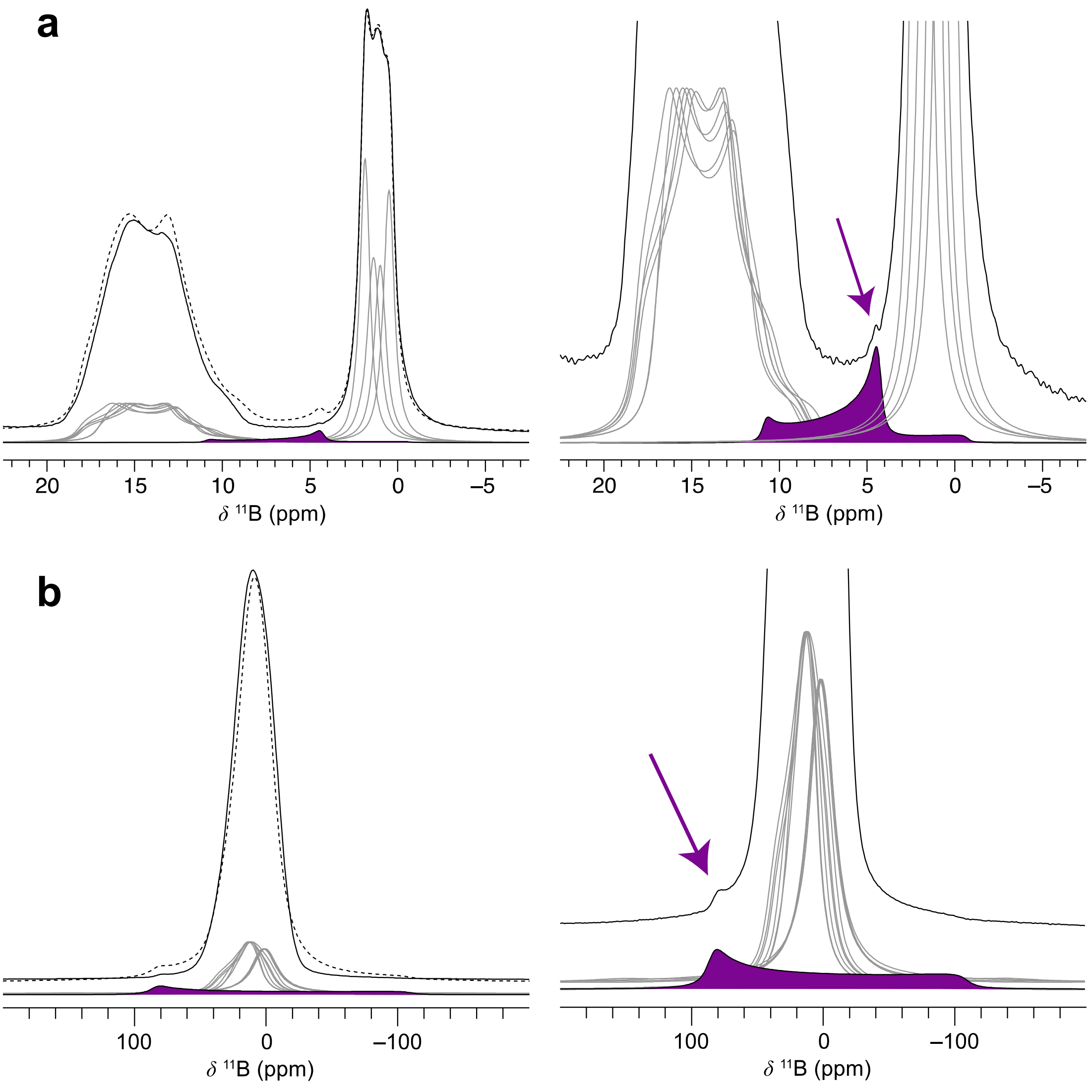

Supplementary Figure 3. ^{11}B MQMAS spectrum of $K_5Ba_2(B_{10}O_{17})_2(BO_2)$ at 9.4 T. The four BO_4 sites are observed and resolved into three distinct sites. BO_3 and BO_2 sites, with their larger quadrupolar coupling constants, are not observed here due to low excitation efficiency and low relative intensity (11 distinct B sites).[4,5] The spectrum was recorded using a z-filtered pulse sequence with excitation and conversion pulses of 9.0 and 3.0 μs followed by a 33 μs selective pulse.[6] Acquisition in the indirect dimension comprised 800 t_1 increments of 20.833 μs. For each t_1-slice, 144 scans were averaged and the recycle delay was 0.5 s, resulting in an experimental time of 16 hours. See Supplementary Figure. 4 for higher field MQMAS. Inset: 3D image of the same, highlighting the 1:2:1 ratio of peak intensities.

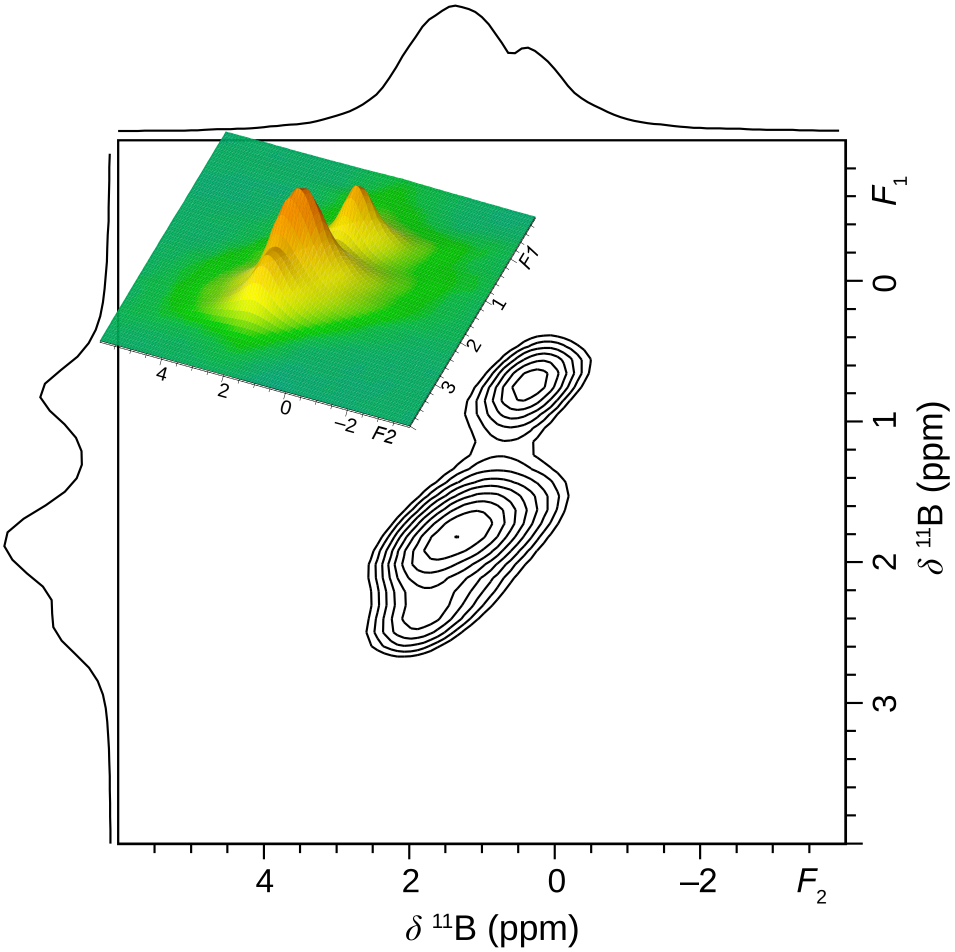

Supplementary Figure 4. a Experimental and **b** simulated ^{11}B MQMAS spectra of $K_5Ba_2(B_{10}O_{17})_2(BO_2)$ at 16.4 T. The experimental spectrum was recorded using a split-t_1 whole-echo pulse sequence with excitation and conversion pulses of 7.0 and 2.25 µs followed by a 24.8 µs selective pulse[7,8]. Acquisition in the indirect dimension comprised 128 t_1 increments of 142.22 µs. For each t_1-slice, 480 scans were averaged and the recycle delay was 1.0 s, resulting in an experimental time of 17 h. Negative contours in **a** reflect t_1 artefacts due to the high intensity of the BO_4 resonances relative to the BO_3 resonances. The low intensity BO_3 resonances are distorted due to non-uniform MQ excitation across the second-order quadrupolar-broadened central transition.[5] Neither the experimental nor simulated spectra showed evidence of the BO_2 resonance which should be centered around 5 ppm in the F_2 dimension; this is attributed to the low relative intensity of this resonance combined with the much lower MQ excitation of this site due to its larger quadrupolar interaction.[5]

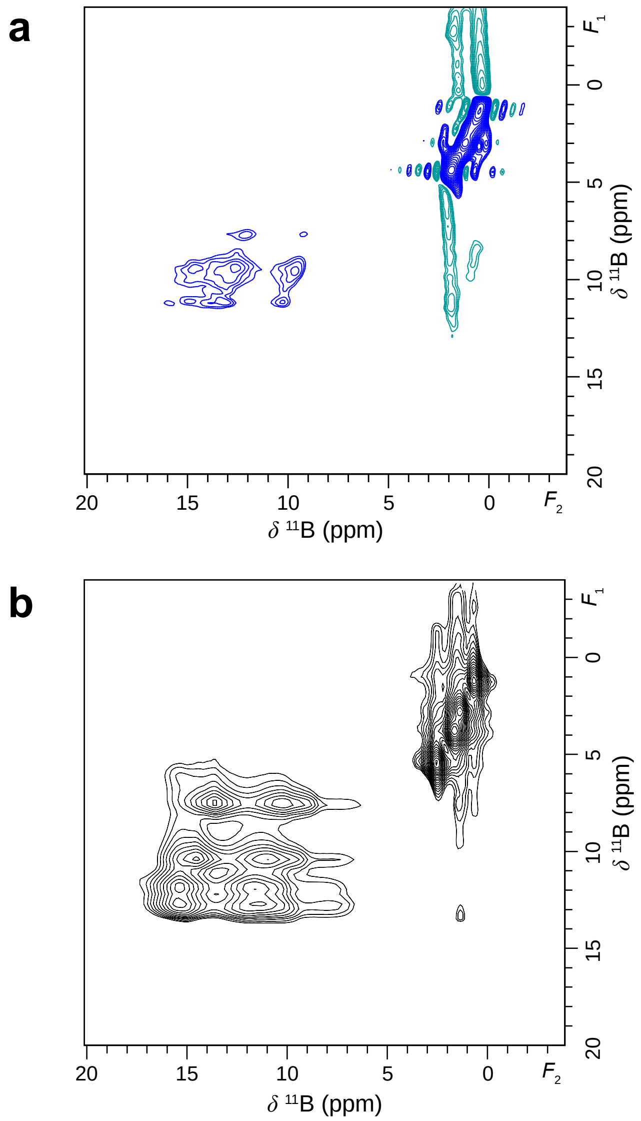

Supplementary Figure 5. ^{10}B NMR static QCPMG spectrum of $K_5Ba_2(B_{10}O_{17})_2(BO_2)$ at 9.4 T. Simulated line shapes for the eleven individual boron sites are shown below the experimental spikelet spectrum and sum of the fits (purple). The experimental and summed spectra are shown at ¾ scale relative to the individual sites.

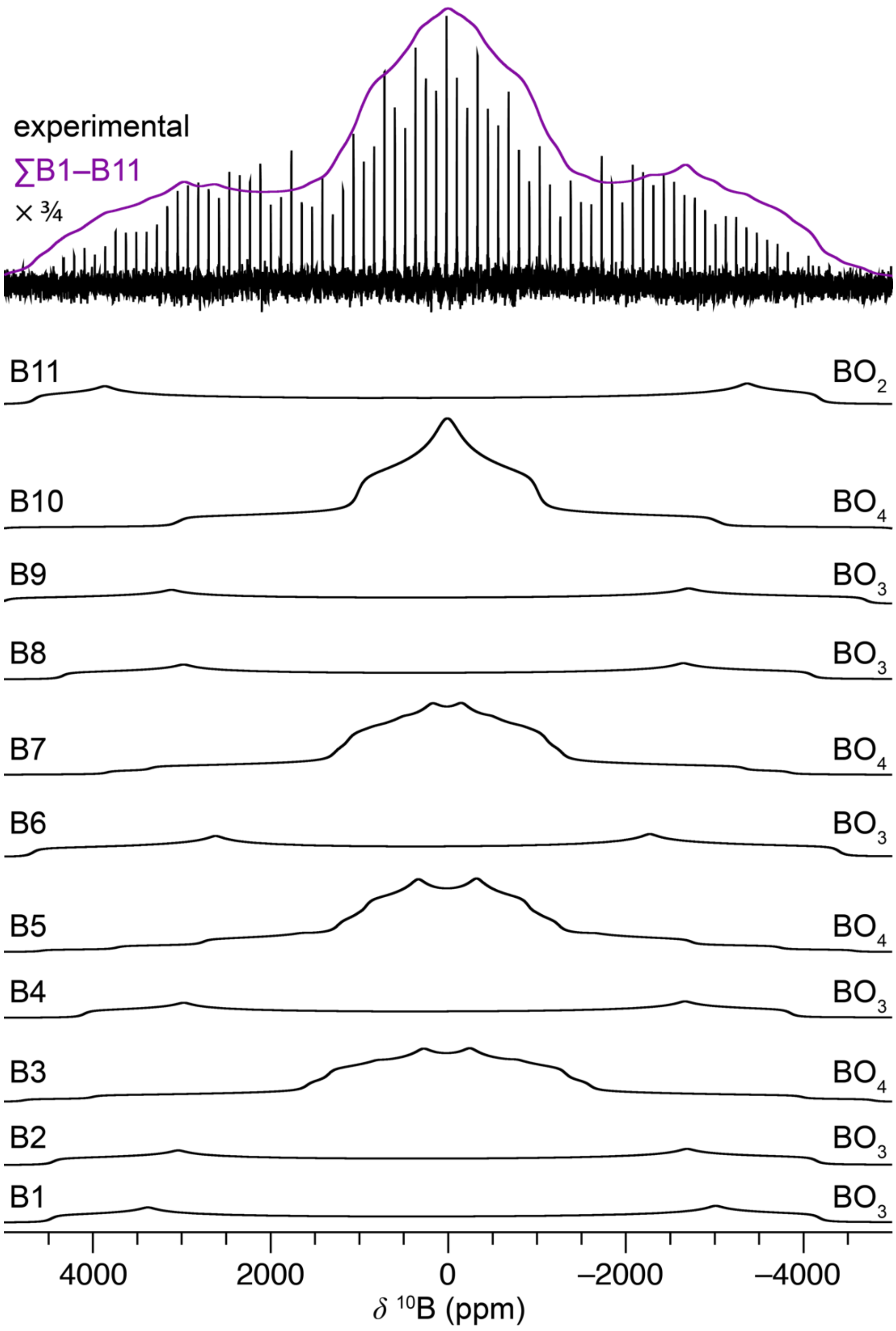

Supplementary Figure 6. Visualization of the **a** ^{11}B shielding tensor and **b** ^{17}O quadrupolar coupling tensor overlaid on the partial structure of $K_5Ba_2(B_{10}O_{17})_2(BO_2)$ in MagresView.[9] Boron in blue, oxygen in orange, potassium in purple, barium in green. Note the tensor orientation along the O(17)–B(11) axis in the isolated BO_2.

a

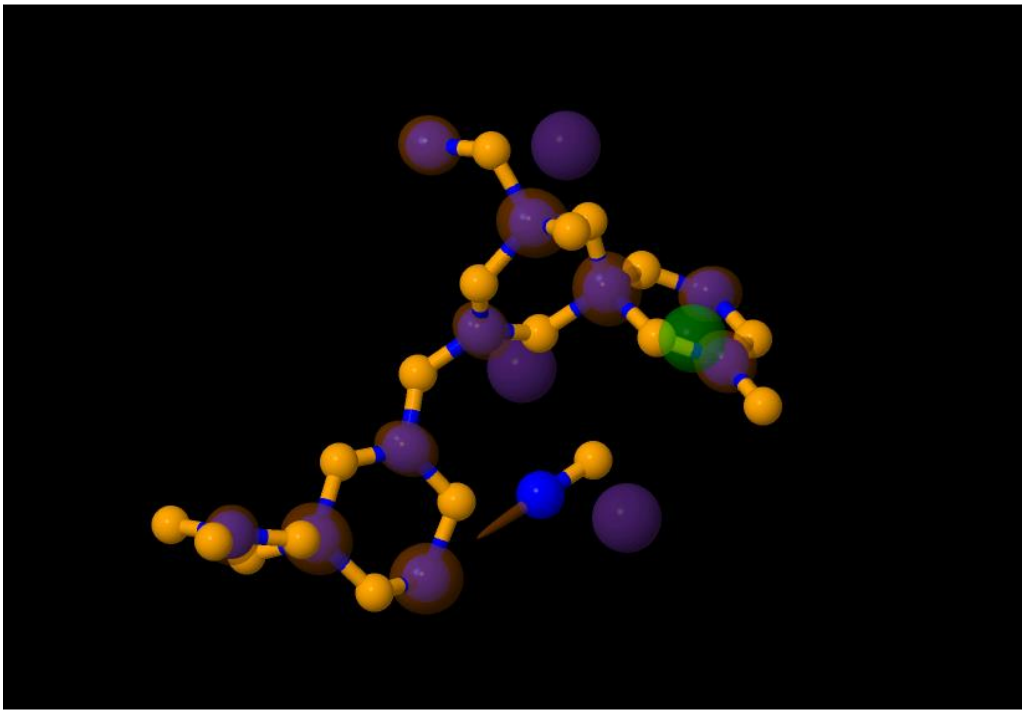

b

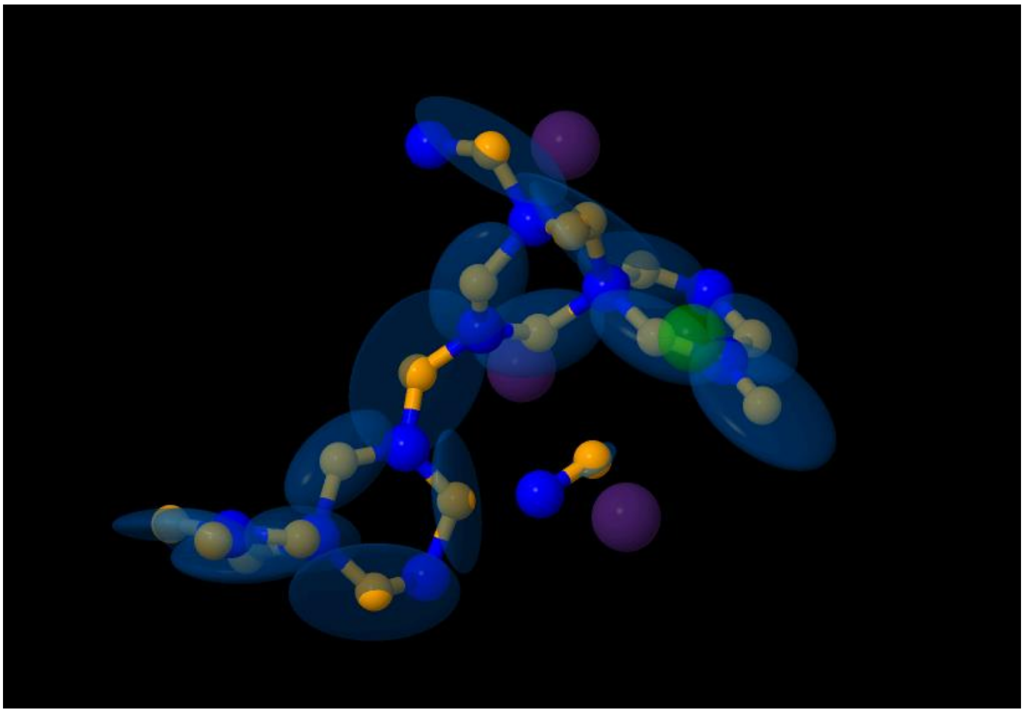

Supplementary Figure 7. Calculated (red) and experimental (black) infrared spectra of $K_5Ba_2(B_{10}O_{17})_2(BO_2)$. The strong bands at 1247, 1334, and 1380 cm^{-1} are mainly due to the asymmetric stretching of the BO_3 units. The bands at 1100, 984 cm^{-1} and 958, 907, 846 cm^{-1} can be assigned to the antisymmetric stretching modes and stretching frequencies of the BO_4 tetrahedra, respectively. The absorption bands around 755 and 780 cm^{-1} can be assigned to both the BO_3 and BO_4 bending modes. Based on the above analysis, the existences of liner BO_2 unit, BO_3 triangles as well as BO_4 tetrahedra are further confirmed. Whereas the bands below 700 cm^{-1} can be assigned to the bending and stretching vibrations of M–O (M = K and Ba) bonds and complex lattice vibrations.

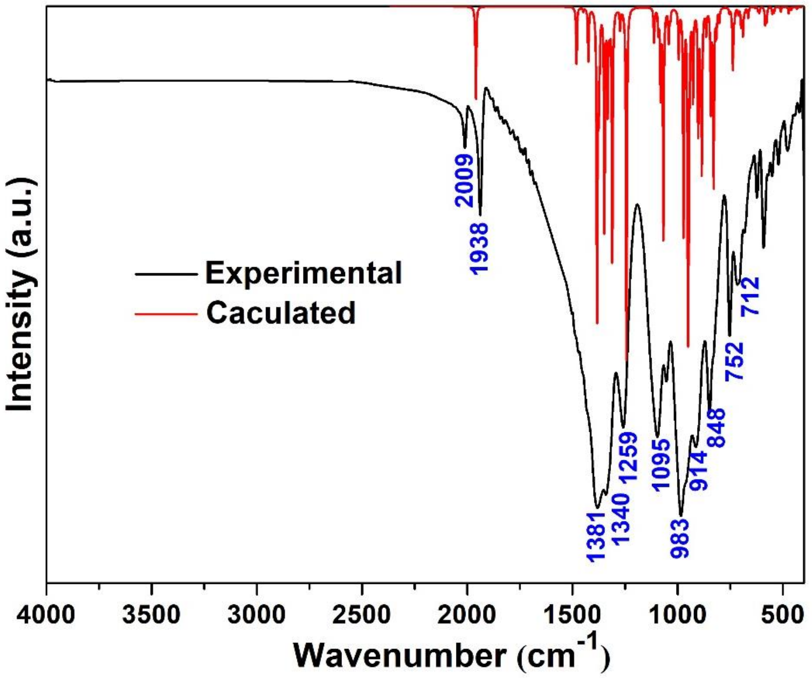

Supplementary Figure 8. TG–DSC curves and XRD patterns of $K_5Ba_2(B_{10}O_{17})_2(BO_2)$. **a** Thermal gravimetric (TG) and differential scanning calorimetry (DSC) curves of $K_5Ba_2(B_{10}O_{17})_2(BO_2)$. **b** Powder X-ray diffraction patterns of $K_5Ba_2(B_{10}O_{17})_2(BO_2)$: calculated and after heated at 700 °C, as well as heated at 740 °C and after melting, respectively. $K_5Ba_2(B_{10}O_{17})_2(BO_2)$ is stable up to 732 °C according to TG–DSC curves (Supplementary Figure 8a) and powder XRD analysis (Supplementary Figure 8b). As the temperature increases, the title compound decomposes endothermically (endo) into other phases (majority $K_5B_{19}O_{31}$, PDF#52-0259) and then melts at a temperature of 776 °C. The analysis of the powder XRD pattern of the solidified melt reveals that the solid product exhibits a different diffraction pattern from the initial $K_5Ba_2(B_{10}O_{17})_2(BO_2)$ powder, which indicates that $K_5Ba_2(B_{10}O_{17})_2(BO_2)$ melts incongruently and that a suitable flux should be introduced to decrease the temperature during crystal growth.

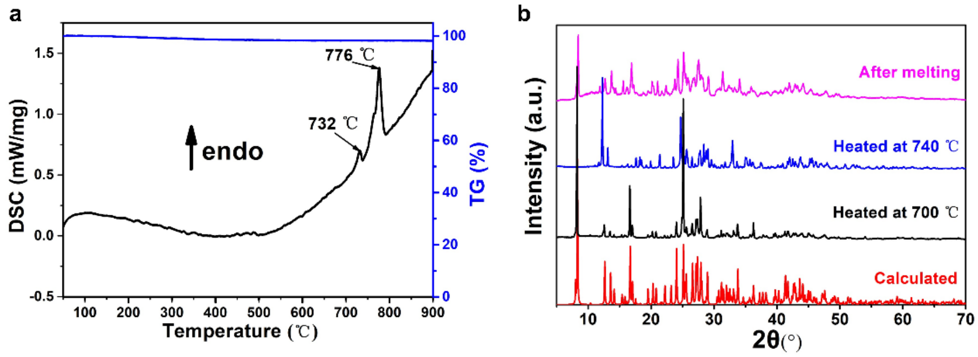

Supplementary Figure 9. The UV−vis−NIR spectrum of $K_5Ba_2(B_{10}O_{17})_2(BO_2)$.

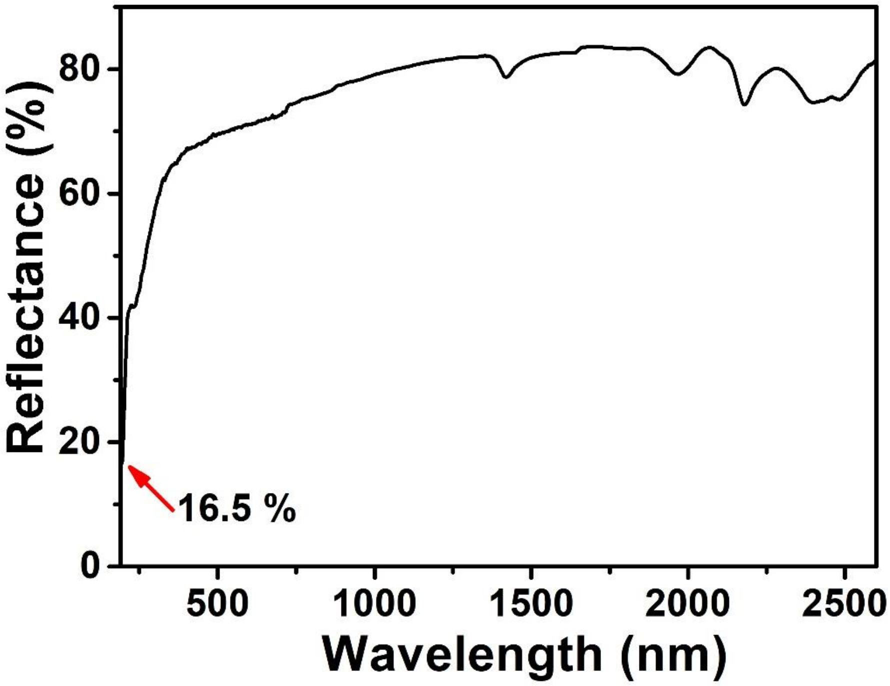

Supplementary Figure 10. Band structure of $K_5Ba_2(B_{10}O_{17})_2(BO_2)$ calculated with GGA-PBE.

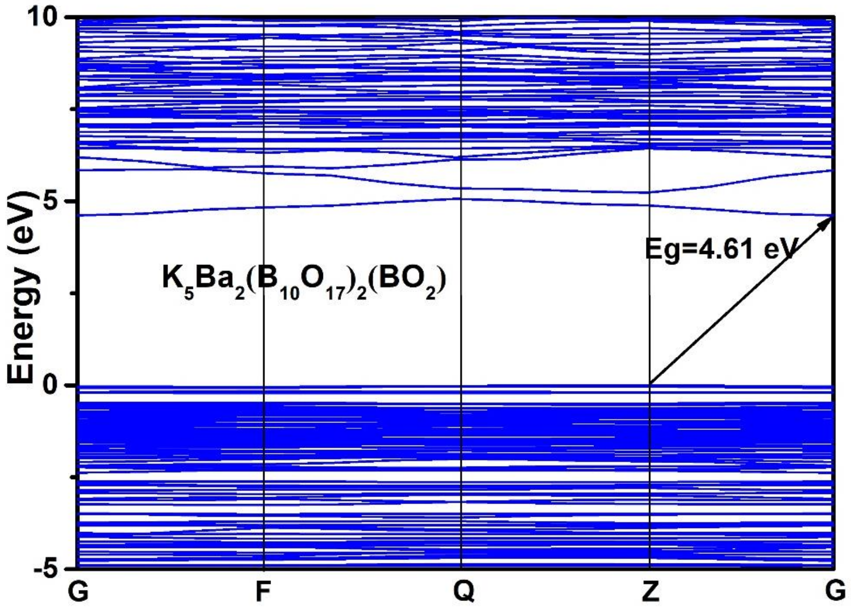

Supplementary Figure 11. Density of states of $K_5Ba_2(B_{10}O_{17})_2(BO_2)$ calculated with GGA-PBE.

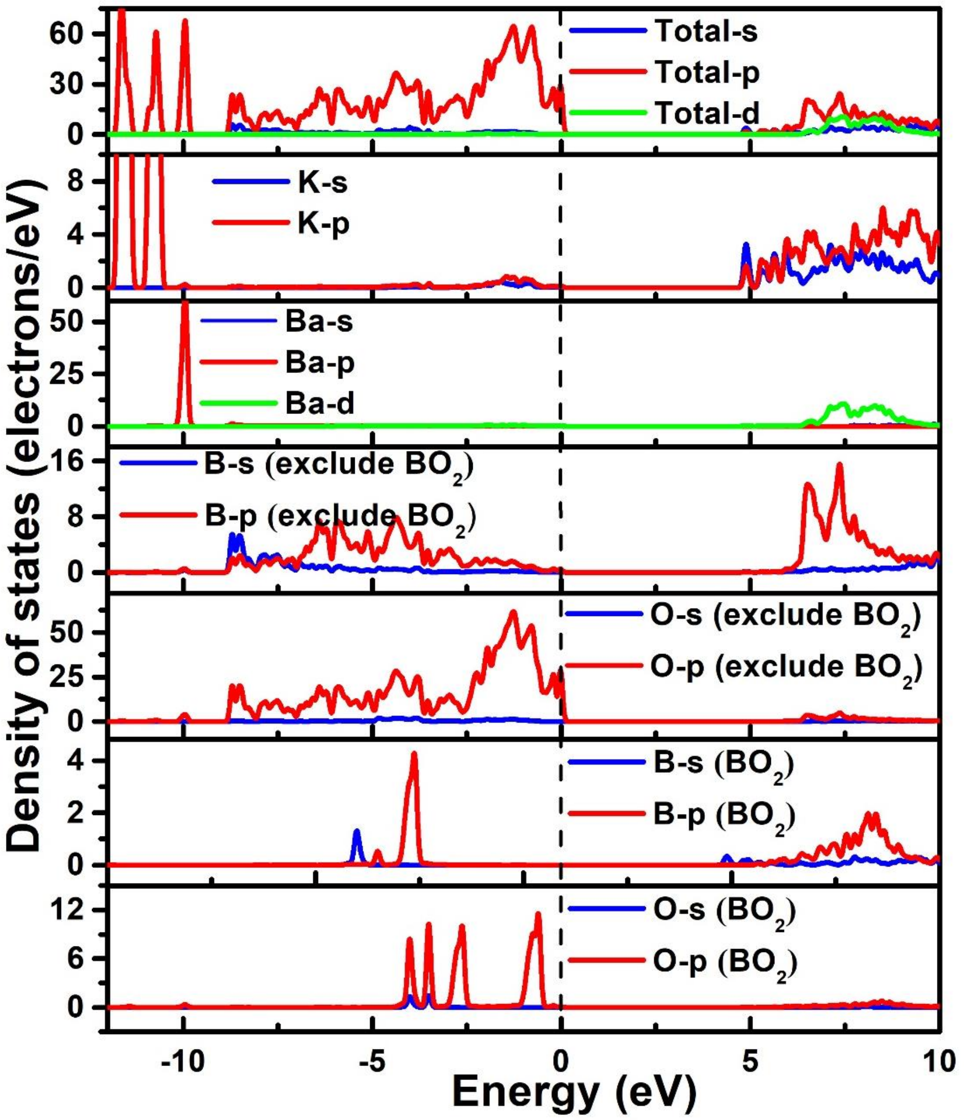

Supplementary Figure 12. Band structures of $K_5Ba_2(B_{10}O_{17})_2Cl$ and $K_5Ba_2(B_{10}O_{17})_2Br$ calculated with GGA-PBE.

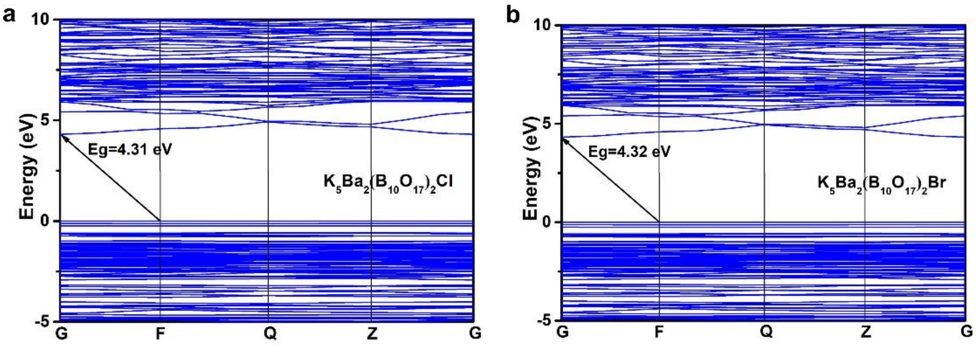

Supplementary Figure 13. Calculated birefringence of $K_5Ba_2(B_{10}O_{17})_2X$ (X = BO_2, Cl, Br).

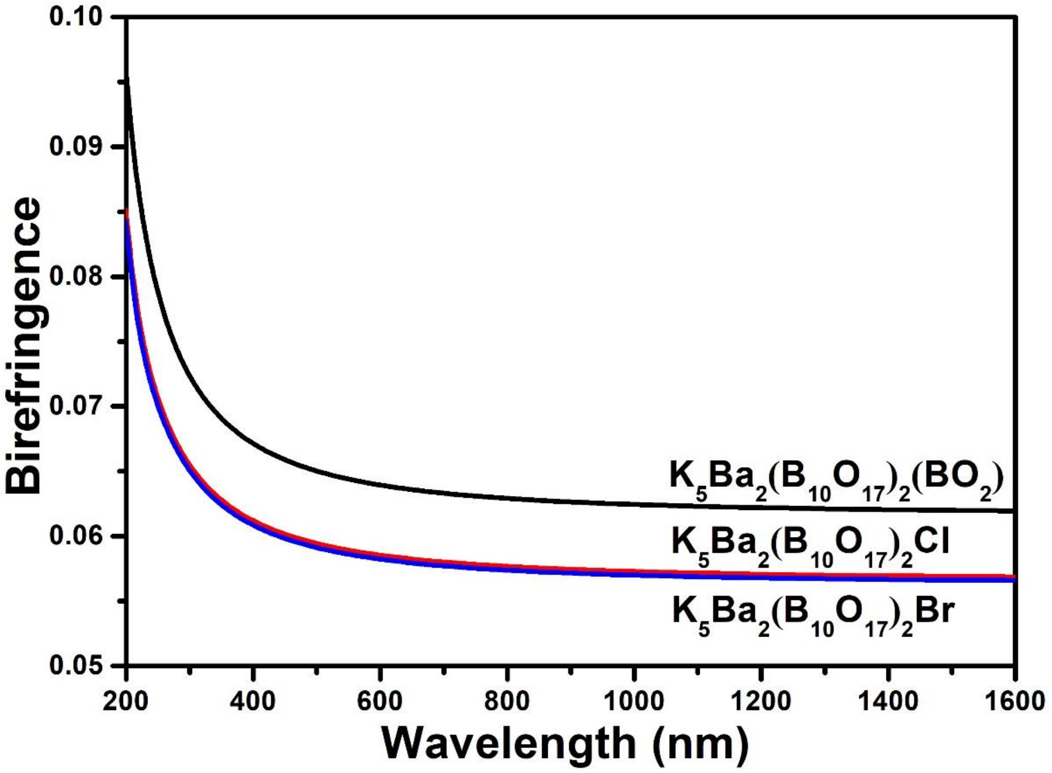

Supplementary References

1. Chen, B. et al. Correlation of network structure with devitrification mechanism in lithium and sodium diborate glasses, *J. Non. Cryst. Solids* **356**, 2641–2644 (2010).
2. Paterson, A. L., Zwanziger, U. W. & Zwanziger, J. W. Network connectivity and crystallization in the transparent ferroelectric nanocomposite $LaBGeO_5$. *J. Phys. Chem. C* **123**, 11860–11873 (2019).
3. Middlemiss, D. S., Blanc, F., Pickard, C. J. & Grey, C. P. Solid-state NMR calculations for metal oxides and gallates: Shielding and quadrupolar parameters for perovskites and related phases. *J. Magn. Reson*. **204,** 1−10 (2010).
4. Zhang, X., Hu, L. & Ren, J. Structural studies of rare earth-doped fluoroborosilicate glasses by advanced solid-state NMR. *J. Phys. Chem. C.* **124**, 8919–8929 (2020).
5. Hung, I. & Gan, Z. Low-power STMAS – breaking through the limit of large quadrupolar interactions in high-resolution solid-state NMR spectroscopy *Phys. Chem. Chem. Phys.* **22**, 21119—21123 (**2020**).
6. Amoureux, J. P., Fernandez, C. & Steuernagel, S. ZFiltering in MQMAS NMR. *J. Magn. Reson. Series A* **123**, 116-118 (1996).
7. Brown, S. P. & Wimperis, S. Two-dimensional multiple-quantum MAS NMR of quadrupolar nuclei. acquisition of the whole echo. *J. Magn. Reson.* **124**, 279-285 (1997).
8. Brown, S. P., Heyes, S. J. & Wimperis, S. Two-dimensional MAS multiple-quantum NMR of quadrupolar nuclei. removal of inhomogeneous second-order broadening. *J. Magn. Reson. Series A* **119**, 280-284 (1996).
9. Sturniolo, S., Green, T. F. G., Hanson, R. M., Zilka, M. Refson, K., Hodgkinson, P., Brown, S. P., Yates, J. R. Visualization and processing of computed solid-state NMR parameters: Magres View and MagresPython *Solid State Nucl. Magn. Reson*. **78**, 64–70 (2016).
